# Low-cesium rice: mutation in *OsSOS2* reduces radiocesium in rice grains

**DOI:** 10.1038/s41598-017-02243-9

**Published:** 2017-05-25

**Authors:** Satoru Ishikawa, Shimpei Hayashi, Tadashi Abe, Masato Igura, Masato Kuramata, Hachidai Tanikawa, Manaka Iino, Takashi Saito, Yuji Ono, Tetsuya Ishikawa, Shigeto Fujimura, Akitoshi Goto, Hiroki Takagi

**Affiliations:** 1 Institute for Agro-Environmental Sciences, National Agriculture and Food Research Organization (NARO), Tsukuba, 305-8604 Japan; 2 Institute of Agrobiological Sciences, NARO, Tsukuba, 305-8604 Japan; 3 Hama Agricultural Regeneration Research Centre, Fukushima Agricultural Technology Centre, Minamisoma, 975-0007 Japan; 4 Fruit Tree Research Centre, Fukushima Agricultural Technology Centre, Fukushima, 960-0231 Japan; 5 Tohoku Agricultural Research Center, NARO, Fukushima, 960-2156 Japan; 6 Institute of Crop Science, NARO, Tsukuba, 305-8518 Japan; 7 Iwate Biotechnology Research Center, Kitakami, 024-0003 Japan; 8 Ishikawa Prefectural University, Ishikawa, 921-8836 Japan

## Abstract

In Japan, radiocesium contamination in foods has become of great concern and it is a primary issue to reduce grain radiocesium concentration in rice (*Oryza sativa* L.). Here, we report a *low-cesium rice mutant 1* (*lcs1*) with the radiocesium concentration in grain about half that in the wild-type cultivar. Genetic analyses revealed that a mutation in *OsSOS2*, which encodes a serine/threonine-protein kinase required for the salt overly sensitive (SOS) pathway in plants, is responsible for the decreased cesium (Cs) concentrations in *lcs1*. Physiological analyses showed that Cs^+^ uptake by *lcs1* roots was significantly decreased under low-potassium (K^+^) conditions in the presence of sodium (Na^+^) (low K^+^/Na^+^). The transcript levels of several K^+^ and Na^+^ transporter genes, such as *OsHAK1*, *OsHAK5*, *OsAKT1*, and *OsHKT2;1* were significantly down-regulated in *lcs1* grown at low K^+^/Na^+^. The decreased Cs^+^ uptake in *lcs1* might be closely related to the lower expression of these genes due to the K^+^/Na^+^ imbalance in the *lcs1* roots caused by the *OsSOS2* mutation. Since the *lcs1* plant had no significant negative effects on agronomic traits when grown in radiocesium-contaminated paddy fields, this mutant could be used directly in agriculture for reducing radiocesium in rice grains.

## Introduction

Because of the incident at Tokyo Electric Power Company’s Fukushima Daiichi Nuclear Power Plants (TEPCO-FDNPP), which was triggered by the Great East Japan Earthquake and the subsequent tsunami on March 11, 2011, a large amount of radiocesium was dispersed into the surrounding environment^1, 2^. Since radiocesium has a relatively long half-life (^134^Cs, 2.06 years; ^137^Cs, 30.2 years) and enters easily through the food chain into the human body^3, 4^, we must pay special attention to the possible contamination of rice (*Oryza sativa* L.), a staple crop in Japan. To restrict shipments of rice with radiocesium levels over 100 Bq kg^−1^, which is stipulated by the Food Sanitation Act in 2012, all rice produced in Fukushima Prefecture has been inspected for radiocesium concentration^5^. The Ministry of Agriculture, Forestry and Fisheries of Japan announced that no rice with a radiocesium level over 100 Bq kg^−1^ was produced in 2015^6^. From the viewpoint of the potential risk to human health from chronic exposure to radiocesium, however, customers still have great concern about radiocesium concentrations in rice grains. In addition, revitalization of the area where planting is currently restricted is still incomplete.

Depending on the radiocesium concentrations in the soil, several options are recommended for decontamination: topsoil removal, fine-textured topsoil removal using water, and topsoil burying^2, 7^. On the other hand, many reports revealed that application of potassium (K^+^) fertilizer is very effective and more practical in reducing radiocesium uptake by paddy rice^7, 8^. Kato *et al*. recommended to increase the concentration of exchangeable soil K^+^ to 200 mg kg^−1^ soil before planting to reduce the radiocesium concentration in rice grains to the acceptable level^8^. Although K^+^ application is widely implemented in the low-contaminated area in the Fukushima Prefecture, K^+^ must be applied over a long period of time due to the long half-life of ^137^Cs. Continuous application of K^+^ is a laborious and costly countermeasure, and therefore rice cultivars that accumulate less radiocesium in their grains have been proposed.

It is well documented that Cs^+^ is taken up by plants through K^+^ transport pathways because two ions have similar chemical properties^3, 4, 9^. The effectiveness of K^+^ supply can be explained by a competitive effect of K^+^ on root Cs^+^ uptake, and K^+^ transporters and K^+^ channels are suggested to mediate Cs^+^ uptake in roots^3, 9^. In *Arabidopsis*, high affinity K^+^ transporter AtHAK5 is a major mediator of Cs^+^ uptake in roots under K^+^ starvation because the mutant lacking *AtHAK5* accumulates Cs^+^ in whole seedlings at about half the concentration in wild-type plants^10^. Under K^+^-replete conditions, Cs^+^ is likely to be transported into *Arabidopsis* roots via cyclic nucleotide–gated channels (AtCNGCs)^4^. *AtCNGC1* is reported to be a candidate gene for determining the natural variation in shoot Cs concentrations^11^.

There is a substantial genotypic variation in Cs concentrations in rice grains^12, 13^ and stable ^133^Cs concentrations are generally lower in grains of *japonica* cultivars than in those of *indica* cultivars^12^. Significant positive correlation between the concentrations of stable ^133^Cs and radiocesium (^134^Cs and ^137^Cs) in grains was found when 85 cultivars were grown in a paddy field located in Fukushima Prefecture, suggesting that the behavior of radiocesium within rice plants is almost identical to that of stable Cs^13^. The physiological characteristics of Cs^+^ transport in rice have been well studied using radioactive or stable Cs^+ ^^14–16^; however, the genetic mechanisms involved in Cs accumulation in rice are still unknown.

A mutant library can be used not only for forward genetics analysis to identify the genes responsible for Cs accumulation in rice but also as breeding material to produce a practically useful low-Cs-accumulating cultivar. We have previously screened a mutant library produced from Koshihikari (the most popular rice cultivar in Japan) by carbon-ion-beam irradiation, and identified mutants with a nearly undetectable level of cadmium (Cd) in grains^17^. Forward genetic analysis showed that the low-Cd mutants had a mutation in *OsNramp5*, which encodes a manganese transporter in rice. One of the mutants, line *lcd-kmt2* with no agriculturally or economically adverse traits has been registered as a new cultivar, Koshihikari Kan No.1; it is now being introduced into Japanese paddy fields^18^. This strategy can be applied to produce practically useful rice cultivars that accumulate less radiocesium in their grains.

In this study, we identified a *low-cesium rice mutant 1* (*lcs1*) with a deletion in *Os* *SOS2*, which has a significantly lower radiocesium concentration in its grains than the parent cultivar Koshihikari. The serine/threonine protein kinase SOS2 is required for the salt overly sensitive (SOS) regulatory pathway involved in plant salt tolerance^19, 20^. In *Arabidopsis thaliana*, the SOS pathway comprises SOS1, SOS2, and SOS3; SOS2 is activated by SOS3, and the SOS2–SOS3 complex phosphorylates and activates SOS1, a plasma membrane Na^+^/H^+^ antiporter, under salinity^21–23^. Rice also uses the SOS pathway to avoid salt toxicity^24^. However, there are no reports of the involvement of SOS2 in Cs accumulation in plants. We also analyzed the physiological mechanism underlying the decrease in Cs concentration by the *SOS2* mutation in rice.

## Results

### *lcs1* is a practically useful rice mutant with reduced radiocesium uptake

A mutant library of Koshihikari was produced by ion-beam irradiation^17^ and the *lcs1* mutant was selected from 2710 M_2_ (second generation after mutagenesis) plants (Supplementary Fig. S1). The grain ^133^Cs concentration was 0.015 mg kg^−1^ in *lcs1* and 0.05 mg kg^−1^ in the Koshihikari parent (WT). The *lcs1* mutant and WT were cultivated together in three paddy fields about 60 km northwest from TEPCO-FDNPP. Radiocesium concentration in soil was 1057–1705 Bq kg^−1^ for ^134^Cs and from 3894–6387 Bq kg^−1^ for ^137^Cs; for both isotopes, it was highest at site C (Supplementary Table S1). The exchangeable soil Na^+^ concentrations were similar among the sites. The exchangeable soil K^+^ concentration and the K^+^/Na^+^ ratios were lowest at site C and highest at site A. The exchangeable soil K^+^ concentration at all sites was lower than the recommended range (80–250 mg K^+^ kg^−1^) for lowland paddy soil^8^.

**Figure 1:**
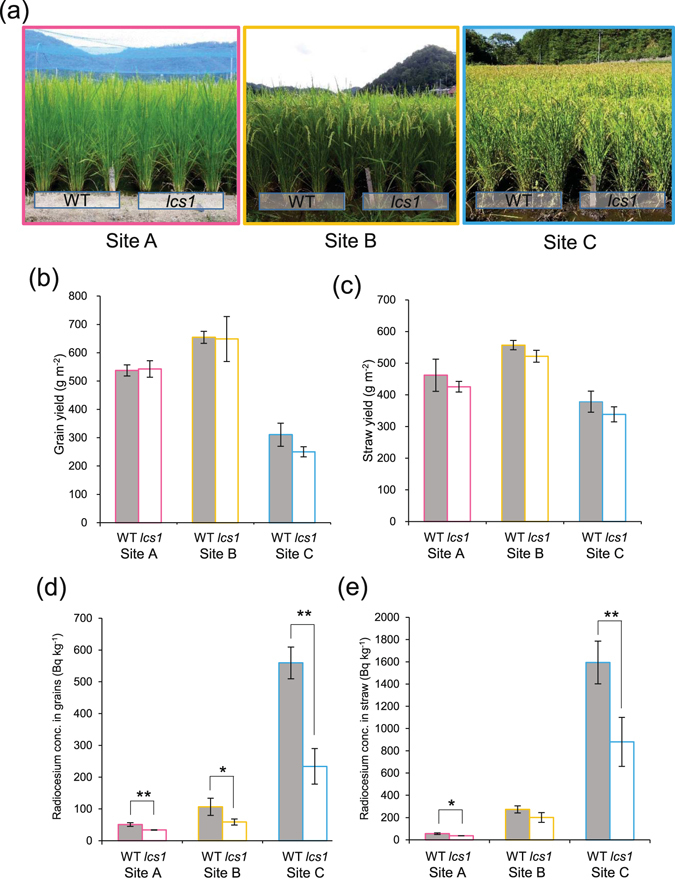
Plant morphology, grain yield, straw yield, and total radiocesium (sum of ^134^Cs and ^137^Cs) in wild-type Koshihikari and its low-cesium mutant (*lcs1*) grown in three paddy fields located in Fukushima Prefecture. (**a**) Morphology of WT and *lcs1* plants grown in three fields (sites A, B, and C). (**b**) Grain yield. (**c**) Straw yield. (**d**) Total radiocesium concentration in grains (unpolished rice). (**e**) Total radiocesium concentration in straw. Data are means ± SD of three biological replicates. **P < 0.01, *P < 0.05 (*t*-test).

The morphology of *lcs1* and WT plants was indistinguishable at all three sites (Fig. 1a). Although grain yields varied among the sites, the difference between *lcs1* and WT was not significant (Fig. 1b). Straw yield was insignificantly lower in *lcs1* than in WT at all sites (Fig. 1c). Total radiocesium (^134^Cs and ^137^Cs) concentration in rice grains varied greatly among the sites and between WT and *lcs1* (Fig. 1d). It was the highest at site C, where radiocesium concentration in *lcs1* was 32% of that in WT. At site B, it exceeded 100 Bq kg^−1^ in WT, but was almost half the WT level and below the 100 Bq kg^−1^ limit in *lcs1*. At site A, it was the lowest and was significantly lower in *lcs1* than in WT. Radiocesium concentration in straw was also lower in *lcs1* than in WT at all sites, although at site B the difference was not significant (Fig. 1e). These results indicate that *lcs1* is a low-Cs mutant line potentially useful for agriculture.

The concentrations of K^+^, sodium (Na^+^), and rubidium (^85^Rb), which are group I alkali metals, were also compared between the WT and *lcs1* at all sites. The differences in K^+^ concentrations between the WT and *lcs1* were small, although *lcs1* showed significantly lower K^+^ concentrations than the WT in grains at site B and in straw at sites A and B (Supplementary Fig. S2a,b). Na^+^ concentrations in grains and straw were significantly lower in *lcs1* than in WT at site C (Supplementary Fig. S2c,d). Stable Rb concentrations in grains and straw were lower in *lcs1* than in WT at all sites (Supplementary Fig. S2e,f). Thus, the *lcs1* mutation affects the concentrations of not only Cs but also some other alkali elements in plants.

### A mutation in *OsSOS2* is responsible for reducing the radiocesium level in *lcs1*

We developed an F_2_ population (second filial generation) derived from a cross between *lcs1* and the *indica* cultivar Habataki, and performed quantitative trait loci (QTL) analysis. The F_2_ population segregated widely for ^133^Cs and ^85^Rb concentration in grains (Supplementary Fig. S3a,b). QTL analysis revealed that major-effect QTLs for reducing Cs and Rb concentration in *lcs1* grains were co-localized on chromosome 6 (Supplementary Fig. S3c,d). Linkage analysis mapped the *lcs1* candidate locus to 1.64 Mbp between the markers ad06010939 and RM20377 on chromosome 6 (Supplementary Fig. S3e). To identify the gene responsible for the *lcs1* phenotype, we sequenced the whole genomes of WT and *lcs1* and compared the candidate regions on chromosome 6; this analysis identified two mutations (Supplementary Table S2). One of them was a 1485 bp deletion in the gene *Os06g0606000* (Supplementary Fig. S3f), which is annotated as encoding the SALT OVERLY SENSITIVE 2 protein (*OsSOS2*) in the Rice Annotation Project Database (http://rapdb.dna.affrc.go.jp/). The other mutation was an A-to-C SNP transition in an intergenic region, suggesting that the deletion in *OsSOS2* is responsible for low Cs concentration in *lcs1*.

**Figure 2:**
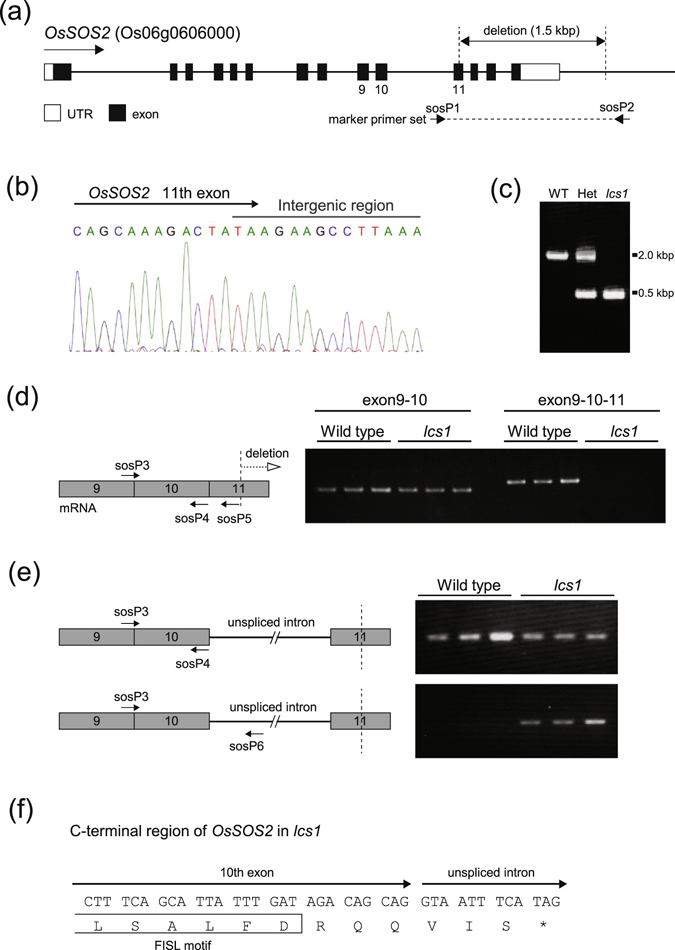
Identification of the gene responsible for the decreased Cs^+^ uptake in *lcs1*. (**a**) Structure of *OsSOS2* (*Os06g0606000*) and the mutation site in *lcs1*. (**b**) Genomic sequence of the mutation site: exon 11 of *OsSOS2* is joined to the intergenic region. (**c**) Fragments of the genomic region containing the mutation site amplified using the marker primer set sosP1and sosP2 shown in (**a**). Het, an F_1_ plant derived from a cross between WT and *lcs1*. (**d**) RT-PCR analyses of *OsSOS2* transcripts using the primers sosP3, sosP4, and sosP5 designed to recognize exons 9–11. RNA was extracted from the whole roots of 8-day-old seedlings. (**e**) RT-PCR analysis of transcripts using the primers sosP3, sosP4, and sosP6. (**f**) Codons and the amino acid sequence around the junction between exon 10 and the unspliced intron of *OsSOS2* in *lcs1*. The FISL motif is a site for SOS3 binding.

As shown in Fig. 2a, *OsSOS2* in the *lcs1* mutant lacked part of exon 11 and all the downstream exons (12–14). Genomic sequence data revealed that truncated exon 11 was joined to the intergenic region (Fig. 2b). Polymerase chain reaction (PCR) was conducted to amplify the mutated genomic region using a marker primer set (sosP1 and sosP2) designed to recognize the regions flanking the deletion site in *lcs1* (Fig. 2a). The sizes of WT and *lcs1* PCR fragments were clearly different and consistent with the 1458-bp deletion in *lcs1* (Fig. 2c). Reverse transcriptase (RT)-PCR analysis detected a transcript with exons 9 and 10 in *lcs1* (Fig. 2d), but no transcripts that would include exons 9–11, although the annealing sites for the primers sosP3 and sosP5 were present in the *lcs1* genome. Instead, an abnormal transcript, in which the intron between exons 10 and 11 was not spliced out, was abundant in *lcs1* (Fig. 2e). In this transcript, a stop codon appeared immediately downstream of exon 10 (Fig. 2f). These results strongly suggest that *OsSOS2* protein molecules in *lcs1* are C-terminally truncated.

### Low Cs^+^ uptake in *lcs1* requires the presence of Na^+^ under low-K^+^ conditions

Since the *sos2* mutant of *A*. *thaliana* is hypersensitive to NaCl^20^, we examined whether *lcs1* is less tolerant to high-Na^+^ stress than is WT. No difference in growth was found between WT and *lcs1* seedlings grown on a nutrient solution without Na^+^ (Supplementary Fig. S4). When the plants were treated with 100 mM NaCl for 5 days, the *lcs1* leaves rolled up and yellowed (Supplementary Fig. S4a), and the dry weight of shoots and roots became significantly lower in *lcs1* than in WT (Supplementary Fig. S4b,c). This result suggests that the *OsSOS2* mutation in rice increased sensitivity to NaCl.

**Figure 3:**
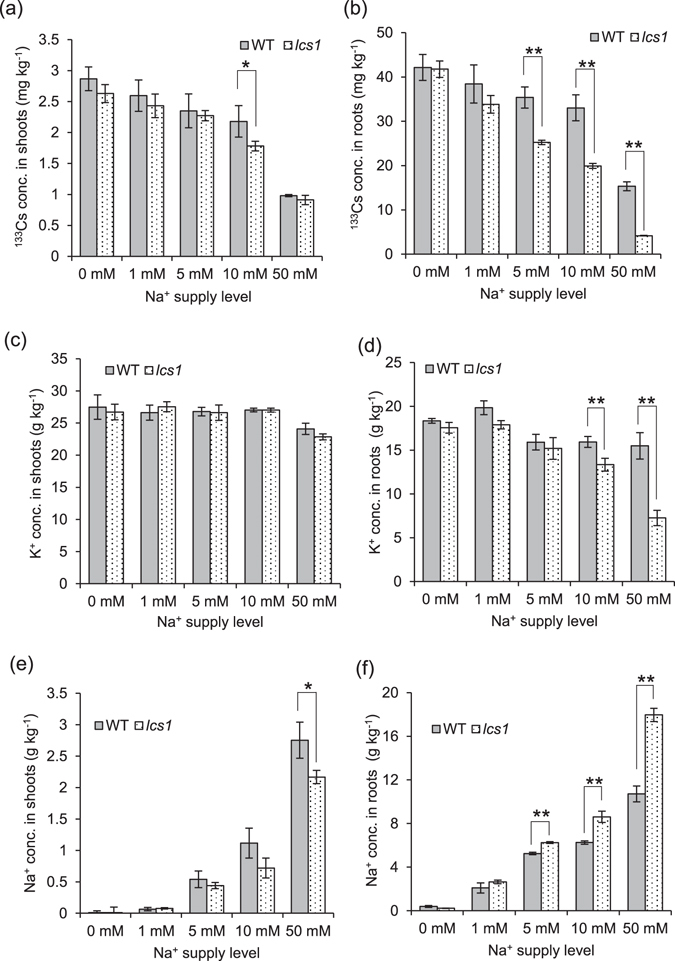
Effects of varying levels of Na^+^ on the concentrations of stable ^133^Cs, K^+^, and Na^+^ in the shoots and roots of WT and *lcs1*. (**a,b**) ^133^Cs concentrations in (**a**) shoots and (**b**) roots. (**c,d**) K^+^ concentrations in (**c**) shoots and (**d**) roots. (**e,f**) Na^+^ concentrations in (**e**) shoots and (**f**) roots. Plants pre-cultured in 0.2 mM K^+^ for 7 days were treated with nutrient solution containing 0.1 µM Cs^+^ and low K^+^ (0.05 mM) at varying levels of Na^+^ for 24 h. Data are means ± SD of three biological replicates. **P < 0.01, *P < 0.05 (*t*-test).

To analyze the physiological mechanism underlying the decrease in Cs accumulation in *lcs1*, we mimicked the difference between WT and *lcs1* in long-term radiocesium accumulation in the field by performing short-term hydroponic experiments. Seedlings were grown in half-strength Kimura B solution, which is widely used for rice^25^. K^+^, Na^+^, and stable ^133^Cs^+^ levels in the nutrient solution were selected in our preliminary experiments with reference to published data^26–28^. The ^133^Cs^+^ level (0.1 μM) was set as low as possible. First, we examined ^133^Cs^+^ uptake in WT and *lcs1* at different Na^+^ levels for 24 h (Fig. 3). ^133^Cs concentrations gradually decreased with increasing external Na^+^ levels; they were significantly lower in *lcs1* than in WT at ≥5 mM Na^+^ in roots (Fig. 3b), but only at 10 mM Na^+^ in shoots (Fig. 3a). Na^+^ did not affect shoot K^+^ concentrations (Fig. 3c), but those in roots were significantly lower in *lcs1* than in WT at 10 and 50 mM Na^+^ (Fig. 3d). Na^+^ concentrations gradually increased with increasing external Na^+^ levels; they were significantly higher in *lcs1* than in WT at ≥5 mM Na^+^ in roots (Fig. 3f) but tended to be lower in *lcs1* than in WT in shoots, with a significant difference at 50 mM Na^+^ (Fig. 3e). These data indicate that the decreased Cs^+^ uptake by *lcs1* roots is linked to an increase in root Na^+^ concentrations.

**Figure 4:**
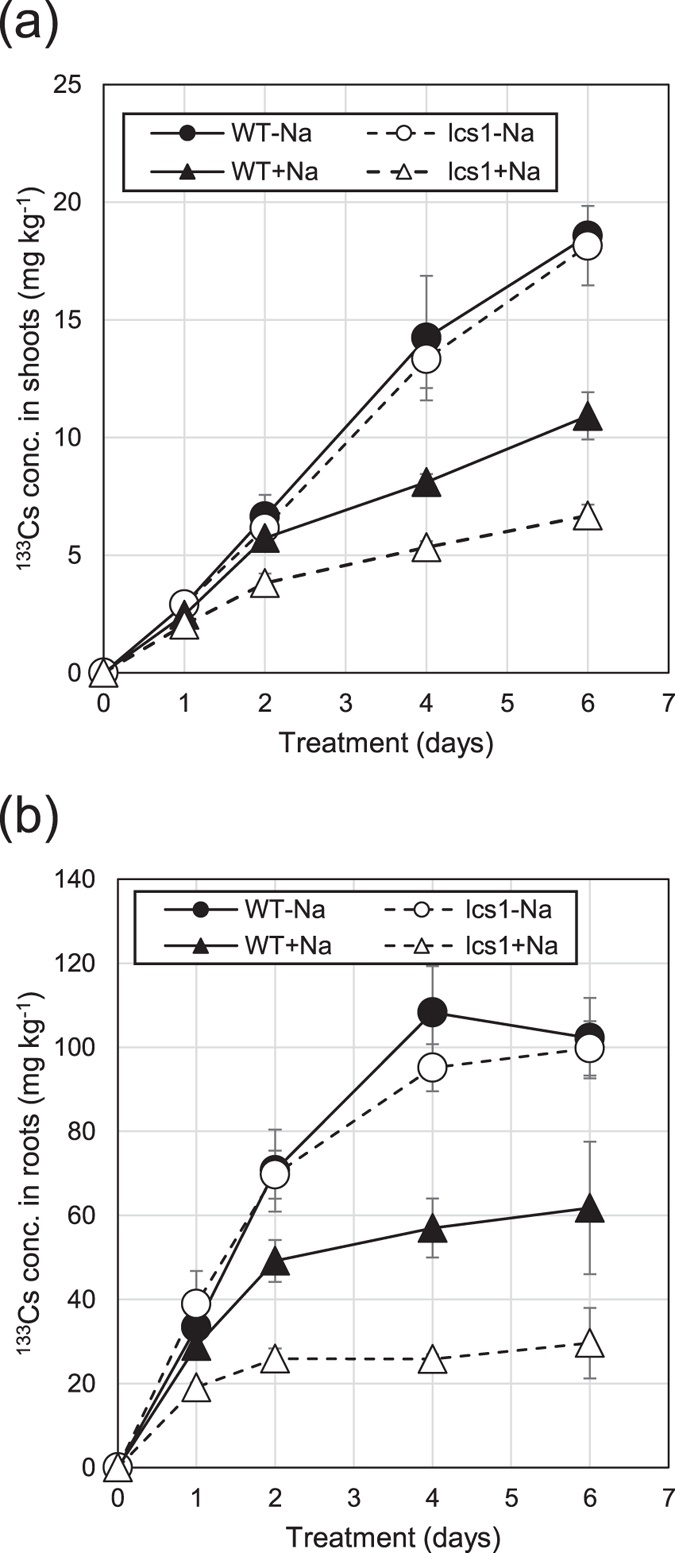
Time course of ^133^Cs concentrations in (**a**) shoots and (**b**) roots of WT and *lcs1*. Plants were treated with nutrient solution containing 0.1 µM Cs^+^ in the presence (+Na^+^) or absence (−Na^+^) of 10 mM Na^+^ under low-K^+^ (0.05 mM) conditions. Data are means ± SD of three biological replicates.

Next, we examined the time course of changes in ^133^Cs concentration in shoots and roots of WT and *lcs1* grown in the presence or absence of 10 mM Na^+^ under low-K^+^ (0.05 mM) conditions (Fig. 4). Our preliminary data showed that the weight of *lcs1* plants did not significantly differ from that of WT plants after a 1-week treatment with 10 mM Na^+^, but brown spots and the yellowing of lower leaves were more noticeable in *lcs1* than in WT. We did not find any differences in Cs concentrations in roots or shoots between the WT and *lcs1* grown in the absence of Na^+^ during the 6-day treatment. Na^+^ supply to low-K^+^ medium markedly decreased Cs concentrations in shoots and roots; Cs concentrations were clearly lower in *lcs1* than in WT. The difference in shoot Cs concentration was observed from day 2 of treatment; shoot Cs concentration in *lcs1* was lower than that in WT by 35% on day 4 and by 40% on day 6 (Fig. 4a). Root Cs concentration was lower in *lcs1* than in WT from day 1 of treatment, and the difference increased thereafter. At day 6, root Cs concentration in *lcs1* was approximately half that of WT (Fig. 4b). Therefore, Na^+^ is essential for suppressing Cs accumulation in *lcs1*.

**Figure 5:**
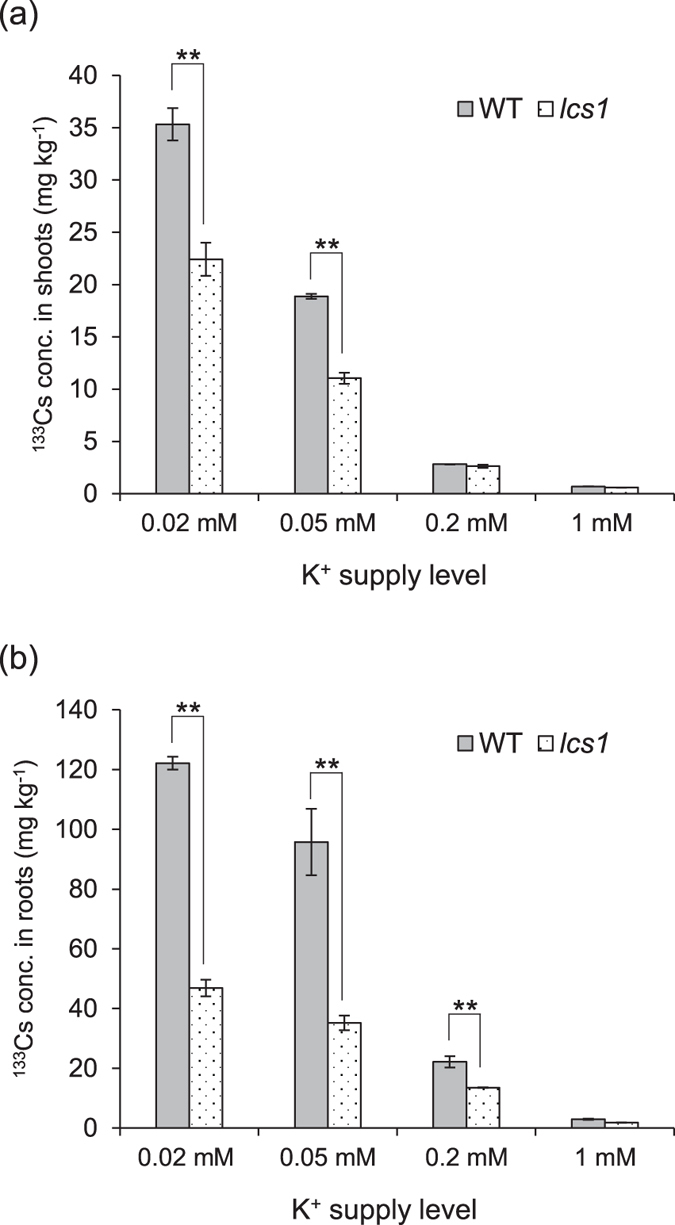
Effects of varying K^+^ levels on ^133^Cs concentrations in (**a**) shoots and (**b**) roots of WT and *lcs1*. Plants were treated with nutrient solution containing 0.1 µM Cs^+^ in the presence of 10 mM Na^+^ and varying levels of K^+^ for 6 days. Data are means ± SD of three biological replicates. **P < 0.01 (*t*-test).

We tested the effect of varying levels of K^+^ on Cs^+^ uptake of plants grown in the presence of 10 mM Na^+^ for 6 days (Fig. 5). In both WT and *lcs1*, ^133^Cs concentrations in roots and shoots decreased remarkably with increasing K^+^ supply, in agreement with the reported inhibitory effect of K^+^ on Cs^+^ uptake by the plants^29–31^. At low K^+^ (0.02 or 0.05 mM) in the presence of Na^+^, root and shoot Cs concentrations were significantly lower in *lcs1* than in WT. At medium K^+^ (0.2 mM) and high K^+^ (1 mM) in the presence of Na^+^, root Cs concentrations were still lower in *lcs1* than in WT, although not significantly so. These results suggest that low external K^+^ and the presence of Na^+^ are necessary for lower Cs^+^ uptake in *lcs1* than in WT.

Similar to the situation with Cs^+^ uptake, ^85^Rb concentrations in shoots and roots were also significantly lower in *lcs1* than in WT when plants were grown for 6 days in nutrient solution containing 10 mM Na^+^ and low K^+^ (0.05 mM) (Supplementary Fig. S5a,b), suggesting that Cs^+^ and Rb^+^ might be transported into rice roots via similar pathways. Taken together, the physiological analyses show that the low-Cs phenotype of *lcs1* appears in the presence of Na^+^ under low-K^+^ conditions.

**Figure 6:**
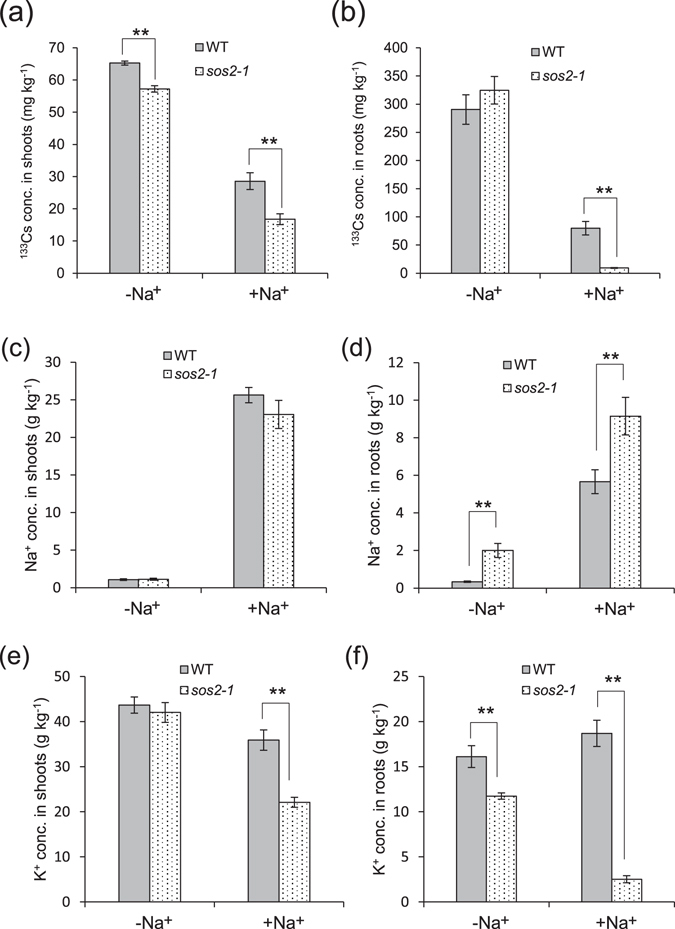
Effect of Na^+^ on the concentrations of ^133^Cs, K^+^, and Na^+^ in the shoots and roots of *Arabidopsis thaliana* Columbia (WT) and *sos2-1* mutant. (**a,b**) ^133^Cs concentrations in (**a**) shoots and (**b**) roots. (**c,d**) Na^+^ concentrations in (**c**) shoots and (**d**) roots. (**e**,**f**) K^+^ concentrations in (**e**) shoots and (**f**) roots. Three-week-old plants grown in MS medium were treated with nutrient solution containing 1 µM Cs^+^, 1 µM Rb^+^ and 10 mM Na^+^ under low-K^+^ (0.05 mM) conditions for 6 days. Data are means ± SD of three biological replicates. **P < 0.01 (*t*-test).

Next, we verified that a mutation in the *A*. *thaliana SOS2* gene inhibits Cs^+^ uptake. The shoot and root ^133^Cs concentrations were significantly lower in *sos2-1* ^21^ than in WT (Columbia) grown in low K^+^ (0.05 mM) and the presence of 10 mM Na^+^ (Fig. 6a,b), especially in *sos2-1* roots, where the decrease in Cs was accompanied by an increase in root Na^+^ concentration (Fig. 6d). No difference in shoot Na^+^ concentration between WT and *sos2-1* was found (Fig. 6c). At Na^+^ supply, K^+^ concentrations in roots and shoots in *sos2-1* were significantly lower than those in WT, indicating that 10 mM Na^+^ severely inhibits K^+^ uptake in the *sos2-1* mutant (Fig. 6e,f). Na^+^ supply had the strongest effect on ^85^Rb concentrations in roots and shoots in *sos2-1* (Supplementary Fig. S5c,d). These results verified the inhibitory effect of *SOS2* mutations on Cs^+^ uptake.

Next, we tested whether the *lcs1* allele of *OsSOS2* confers a low-Cs phenotype in a rice cultivar carrying the wild-type *OsSOS2* allele. We produced an F_2_ population from a cross between *lcs1* and *lcd-kmt2*, a mutant with low-Cd phenotype in the Koshihikari background^17^. In *lcs1* homozygotes, ^133^Cs concentrations in shoots and roots were lower than in WT homozygotes and heterozygotes (Supplementary Fig. S6). This demonstrates that the *lcs1* allele decreases Cs in rice plants. Among 91 F_2_ plants, 5 carried both *lcs1* and *lcd-kmt2* alleles and had decreased concentrations of Cs (Supplementary Fig. S6) and Cd in shoots and roots, indicating that it is possible to produce new cultivars with both traits (low Cs and low Cd).

### Sodium inhibits the expression of several genes encoding monovalent cation transporters in *lcs1*

To investigate the molecular mechanism of decreased Cs^+^ uptake in *lcs1*, we used microarray analysis to compare the root transcriptomes of WT and *lcs1* grown in the presence or absence of 10 mM Na^+^ at 0.05 or 0.2 mM K^+^ for 3 days. Because K^+^ transporters are suggested to mediate Cs^+^ uptake in plants^3, 9^, we focused on the expression of four gene families (*OsHAK*, *OsAKT*, *OsHKT*, and *OsCNGC*) involved in K^+^ transport (Table S3). After excluding the genes with signal values less than 50, we selected four genes (*OsHAK1*, *OsHAK5*, *OsAKT1*, and *OsHKT2;1*) whose down-regulation was stronger in *lcs1* than in WT at low K^+^ in the presence of Na^+^.

**Figure 7:**
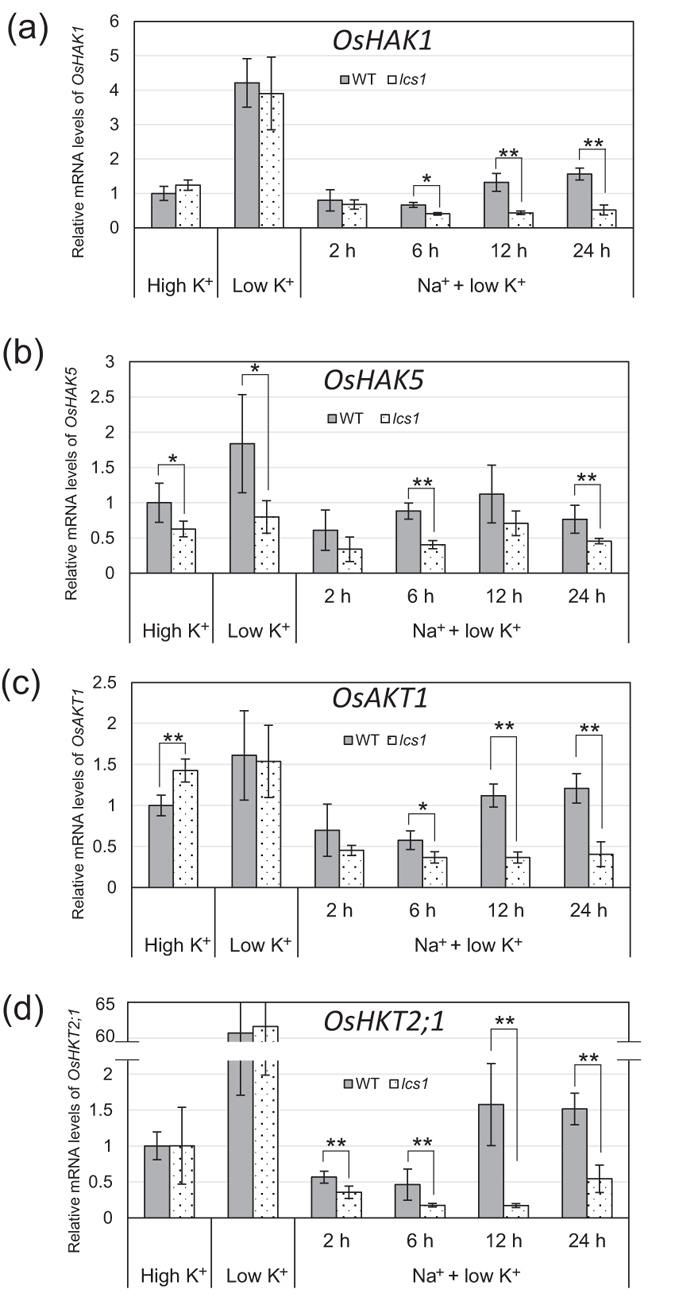
Relative mRNA levels of *OsHAK1* (**a**), *OsHAK5* (**b**), *OsAKT1* (**c**), and *OsHKT2;1* (**d**) in the roots of WT and *lcs1*. Plants were treated with nutrient solution containing 1 mM K (high K^+^) or 0.02 mM (low K^+^) for 7 days. Then the plants grown at low K^+^ were transferred into a solution with 10 mM Na^+^ under low-K^+^ conditions (Na^+^ + low K^+^) and treated for 2, 6, 12, or 24 h. Total RNA was extracted from the roots and the expression of each gene was determined by qRT-PCR. *Actin 1* was used as an internal control. The expression of each gene in wild-type treated with high K^+^ was defined as 1. Data are means ± SD of five biological replicates. **P < 0.01, *P < 0.05 (*t*-test).

Quantitative real-time PCR (qRT-PCR) was conducted to compare the time courses of the expression of *OsHAK1* (Os04g0401700; Fig. 7a), *OsHAK5* (Os01g0930400; Fig. 7b), *OsAKT1* (Os01g0648000; Fig. 7c), and *OsHKT2;1* (Os06g0701700; Fig. 7d) between WT and *lcs1*. In plants pre-grown at low K^+^ (0.02 mM) for 7 days, the expression levels of the four genes were higher than in plants grown at high K^+^ (1 mM). The expression of *OsHKT2;1* was particularly strongly upregulated (>60 times) in K^+^-starved plants. *OsHAK5* was the only gene whose expression was usually lower in *lcs1* than in the WT, irrespective of K^+^ or Na^+^ level. In both WT and *lcs1*, the low K^+^–enhanced expression of the four genes was drastically decreased at 2 h and 6 h after the addition of Na^+^. The expression of all genes was increased at 12 h and 24 h after Na^+^ addition in WT but not in *lcs1*, resulting in significant differences between WT and *lcs1*.

## Discussion

A goal of this study was to provide a practically useful rice line that would accumulate less radiocesium in its grains to improve food safety in Fukushima Prefecture. The *lcs1* mutant was selected from a mutant library of the most popular Japanese rice cultivar Koshihikari (Supplementary Fig. S1). A lower radiocesium concentration in *lcs1* than in WT grains was confirmed in field trials (Fig. 1d). Genetic analysis revealed that *lcs1* had a 1485-bp deletion, which included the sequence encoding the C-terminal region of *OsSOS2* (Fig. 2a, Supplementary Fig. S3f). SOS2 consists of the N-terminal catalytic domain, which was preserved in *lcs1*, and the C-terminal regulatory domain, which was truncated in *lcs1*. The regulatory domain contains a FISL motif, which binds to the calcium-binding protein SOS3, and an adjacent protein phosphatase interaction (PPI) motif^32^. In *lcs1*, the FISL but not the PPI motif was present (Fig. 2f). The PPI motif binds to the type 2 C protein phosphatase ABI2^32^, which may deactivate SOS2 or dephosphorylate its target ion transporters such as SOS1^33^. In the *Arabidopsis abi2-1* mutant, the interaction between ABI2 and SOS2 is disrupted, and this mutant is more tolerant to salt shock than is WT^33^. Mutations in the PPI motif also weaken the SOS2–SOS3 interaction, possibly because of a steric hindrance effect on the neighboring FISL motif^33^. In the *lcs1* mutant, the absence of the PPI motif in *OsSOS2* would prevent its interaction with ABI2 and weaken the *OsSOS2*–OsSOS3 interaction, increasing Na^+^ sensitivity of the mutant (Supplementary Fig. S4) and decreasing Na^+^ extrusion from the roots by OsSOS1. The *lcs1* mutant accumulated more Na^+^ in the roots than did WT (Fig. 3f), which strongly supports this speculation, although shoot Na^+^ concentration was lower in *lcs1* than in WT (Fig. 3e). In *Arabidopsis* shoots, the complex of SOS2 with the calcium sensor CBL10 plays a role in Na^+^ sequestration into the vacuole by regulating tonoplast Na^+^/H^+^ antiporter in shoots and leaves^34, 35^; the *cbl10* mutant contains less Na^+^ in shoots than does WT, and is more sensitive to high Na^+ ^^34^. The *lcs1* mutant might also be defective in vacuolar Na^+^ sequestration in shoots.

SOS2 is well studied as a multifunctional protein kinase that regulates salt tolerance in plants^19–23^; however, to the best of our knowledge, its involvement in plant Cs^+^ uptake has not been documented. SOS2 has no potential transporter domains to mediate Cs^+^ uptake. SOS1 is also unlikely to be related to Cs^+^ influx into the roots because SOS1 is an Na^+^ efflux transporter^23^. The CBL10–SOS2 complex is suggested to regulate a tonoplast Na^+^/H^+^ antiporter in shoots and leaves^34, 35^. These data suggest the involvement of other genes related to Cs^+^ transport together with an alteration in the SOS pathway. Increasing K^+^ supply decreased root Cs^+^ uptake in both WT and *lcs1* (Fig. 5); this indicates that rice Cs^+^ uptake is mediated by a K^+^ transport system^14^. In *Arabidopsis* roots, AtHAK5 mediates K^+^ uptake at external K^+^ concentrations <0.01 mM, whereas AtAKT1 is more important for K^+^ uptake in a range of 0.05–1 mM^36^. In rice, two HAK transporters (OsHAK1^26^ and OsHAK5^37^) and OsAKT1^38^ are crucial for K^+^ uptake at ≤1 mM K^+^. OsHAK5 mediates K^+^ uptake at low K^+^ (0.1 or 0.3 mM), but not at high K^+^ (1 mM)^37^, whereas OsHAK1 and OsAKT1 function in the wide range of 0.05–1 mM K^+ ^^26, 38^. Because significant differences in Cs^+^ uptake between WT and *lcs1* were found at ≤0.2 mM K^+^, OsHAK1, OsHAK5, and OsAKT1 might contribute to the decreased Cs^+^ uptake in *lcs1*. Under K^+^-depleted conditions, high Na^+^ directly represses the expression of *OsHAK1* ^26^ in rice and *AtHAK5* ^28^ in *Arabidopsis*. Na^+^ may affect not only K^+^ uptake but also Cs^+^ uptake through K^+^ transporters. As the first step to clarify the molecular mechanisms of Cs accumulation in WT and *lcs1*, we analyzed the differences in the transcription levels of genes involved in K^+^ transport by microarray and qRT-PCR analyses. The transcript levels of *OsHAK1*, *OsHAK5*, and *OsAKT1* were increased in the roots of *lcs1* and WT grown at low K^+^ (0.02 or 0.05 mM) in the absence of Na^+^ (Fig. 7 and Supplementary Table S3). The expression of *OsHKT2;1*, which encodes the primary Na^+^ transporter in rice under K^+^-starvation conditions^27^, was also remarkably upregulated at low K^+^. However, the time-dependent differences in Cs^+^ uptake (Fig. 4) and the differences in transcript levels (except for *OsHAK5*) were not observed between WT and *lcs1* at low K^+^ alone. In the *lcs1* mutant, the suppression of Cs^+^ uptake into roots and its subsequent translocation to shoots required low K^+^ in the presence of Na^+^ (Figs 3–5). This phenomenon was also observed in the *sos2-1* mutant of *Arabidopsis* (Fig. 6). The transcript levels of *OsHAK1*, *OsHAK5*, *OsAKT1*, and *OsHKT2;1* were significantly lower in *lcs1* roots than in WT roots after adding Na^+^ to low-K^+^ medium (Fig. 7). These results suggest that the K^+^/Na^+^ ratio in medium is a key factor that determines the difference in Cs accumulation between WT and *lcs1*; the decreased Cs^+^ uptake in *lcs1* roots might be caused by suppressed expression of any of these genes.

A rice–barley chimeric HAK1 protein can mediate K^+^, Rb^+^, and Cs^+^ influx in a yeast and has a low capacity to discriminate these ions^39^. OsHAK5 is also likely to mediate Cs^+^ influx in rice roots, especially at low-K^+^ conditions, but the *OsHAK5* transcript level in microarray analysis was substantially lower than that of *OsHAK1* (Supplementary Table S3), and the *OsHAK5* transcript level measured by qRT-PCR was lower in *lcs1* roots than in WT roots irrespective of the supply levels of K^+^ and Na^+^ (Fig. 7b). Therefore, the contribution of OsHAK5 might be smaller than that of OsHAK1, and the decreased Cs^+^ uptake in *lcs1* might be caused by the lower transcript levels of *OsHAK1* rather than *OsHAK5*. In yeast expressing *AtHAK5*, the selectivity for K^+^ over Na^+^ and Cs^+^ was increased by a single point mutation, F130S, which is conserved in HAK1-type and HAK5-type transporters of several species, including OsHAK1^40^. Modification of ion selectivity of a specific transporter (e.g., OsHAK1) might be useful for the production of low-Cs rice without decreasing K^+^ level in the plant.

AtAKT1 and AtHAK5 act as the main system of Rb^+^ absorption in K^+^-starved *Arabidopsis* ^41^, although root Cs^+^ influx is similar in the *akt1* knockout mutant and WT^42^. ^85^Rb concentrations as well as ^133^Cs concentrations in *lcs1* and *sos2-1* mutants were significantly decreased by low K^+^ and the presence of Na^+^ (Supplementary Fig. S5). Because of the similarity of the hydrated ionic radii of Rb^+^ and Cs^+^ (both are 0.329 nm)^43^, some channels and transporters discriminate poorly between them. Kobayashi *et al*. suggested that up to 50% of Cs^+^ uptake in rice roots grown at 0.02 mM K^+^ in the presence of 0.1 μM Cs^+^ is mediated by tetraethyl ammonium–sensitive inward-rectifying K^+^ channels such as OsAKT1^14^. OsAKT1 might mediate Cs^+^ uptake in rice roots, and down-regulation of *OsAKT1* may contribute to the decreased Cs^+^ uptake in *lcs1* grown at a low K^+^/Na^+^ ratio.

Under low K^+^/Na^+^ conditions, Na^+^ substitutes K^+^ to improve rice growth^44^, and OsHKT2;1 is crucial for nutritional Na^+^ uptake into K^+^-starved rice roots^45^. The expression of *OsHKT2;1* was most sensitive to Na^+^ supply at low K^+^ and was significantly lower in the roots of *lcs1* than of WT (Fig. 7d). However, OsHKT2;1 is unlikely to mediate Cs^+^ uptake in rice because of a small permeability to K^+^ and Rb^+^ via OsHKT2;1^27^. The OsCNGC family is also unlikely to be involved in the difference in Cs^+^ accumulation between *lcs1* and WT under low K^+^/Na^+^ conditions because CNGCs mediate Cs^+^ uptake by the roots of K^+^-replete plants^4^. In this study, we could not conclusively identify the K^+^ transporters that are most likely to mediate the suppression of Cs^+^ uptake in *lcs1* grown at low K^+^/Na^+^, and further studies such as post-transcriptional analysis will be necessary to identify these transporters.

Based on the results of hydroponic experiments with seedlings, we analyzed why radiocesium concentrations in *lcs1* grains were lower than in those of WT under the field conditions. Four genes (*OsHAK1*, *OsHAK5*, *OsAKT1*, and *OsHKT2;1*) were expected to be up-regulated in both WT and *lcs1* roots under the field conditions, because exchangeable soil K^+^ levels were lower than the recommended levels (80–250 mg K^+^ kg^−1^)^8^. Although the exchangeable soil Na^+^ levels do not affect rice growth, Na^+^ uptake via OsHKT2;1 must be promoted to substitute for K^+^. Because *lcs1* is unable to extrude Na^+^ from roots, Na^+^ concentration in the roots would gradually increase, and a low K^+^/Na^+^ ratio in roots would down-regulate the levels of *OsHAK1*, *OsHAK5*, and *OsAKT1* transcripts and radiocesium concentrations in *lcs1* grains due to the suppression of radiocesium uptake into the roots. Suppression of the transcript levels of K^+^ transport genes in *lcs1* is a disadvantage for K^+^ uptake, but a large decrease (such as that observed for Cs concentrations) was not found in *lcs1*, probably indicating that K^+^ is rapidly exported from roots to shoots (unlike Cs^+^) to maintain K^+^ homeostasis in the shoots^14^. The exchangeable K^+^/Na^+^ ratio in soil is a key factor that determines the difference in radiocesium concentration in grains between WT and *lcs1*. Significant correlation was observed between the difference in radiocesium concentration (*y*) and the exchangeable K^+^/Na^+^ ratio (*x*) if the former was logarithmically transformed (*y* = 348.51*x* ^−2.402^, *R* ^2^ = 0.913). Lower Na^+^ concentration in grains and straw in *lcs1* at site C, which had the lowest K^+^/Na^+^ ratio among the three sites, can be explained by an alteration in Na^+^ transport systems via OsSOS1 or tonoplast Na^+^/H^+^ antiporter. Although *lcs1* was more sensitive to high-salt stress than was WT in hydroponic experiments (Supplementary Fig. S4), this mutation had no considerable negative effects on plant morphology (Fig. 1a) or grain yield (Fig. 1b) at any of the three field sites. Therefore, the *lcs1* mutant can be directly introduced into paddy fields for reducing radiocesium in rice grains. In fields like site C (high radiocesium concentration and low K^+^/Na^+^ ratio in soil), a combination of K^+^ supply with the use of *lcs1* will be a better method to reduce radiocesium without decreasing grain and straw yield. Moreover, we demonstrated that it is possible to produce rice cultivars with both low-Cs and low-Cd traits (Supplementary Fig. S6). In Japan, there is still a great concern about Cd contamination in rice^17^. We believe that rice cultivars carrying the *lcs1* allele would be an innovative countermeasure to reduce long-term exposure to radiocesium via the food chain.

## Materials and Methods

### Screening for low-Cs mutants

We irradiated seeds of the most popular Japanese cultivar, Koshihikari, with accelerated carbon ions following the method of Ishikawa *et al*.^17^. After irradiation, bulked M_2_ seeds were sowed and about 3000 M_2_ seedlings and 300 WT seedlings were transplanted into plastic pots filled with paddy soil containing 3.0 mg kg^−1^ of ^133^Cs under flooded conditions. The plants were grown in a greenhouse from mid-May to mid-September 2014 at ambient temperature (20–35 °C) under sunlight until grain filling. Grains were harvested from 2710 M_2_ plants and their ^133^Cs concentrations were determined as described below.

### Field experiments

Field experiments were conducted in three paddy fields located 60 km northwest from TEPCO-FDNPP. Bulked soil samples were collected from each site to a depth of 15 cm before planting and used for chemical analysis including radiocesium. M_5_ or M_6_ *lcs1* plants and WT Koshihikari were grown at sites B and C in 2014 and at site A in 2015. Commercial fertilizer was applied before transplanting (60 kg N and 60 kg P_2_O_5_ per ha). Potassium was not applied to avoid competition with radiocesium for uptake by rice roots. At maturing stage, rice was harvested by cutting the stems 10–15 cm above the soil level to prevent contamination with radiocesium from the soil, and separated into grains and straw. Husked grains (brown rice) were sieved through a 1.8-mm mesh to remove immature grains and weighed to evaluate grain yield. After weighing, grain and straw samples were used to analyze the concentration of radiocesium and other metals as described below.

### Radiocesium and metal analysis

For the determination of total ^137^Cs and ^134^Cs, soil, rice grain, and straw samples were placed in a U8 or 2 L Marinelli container and analyzed using a germanium semiconductor detector (GX5520, Canberra Co.)^46^. The concentrations of ^134^Cs and ^137^Cs were corrected to the date of harvest based on radioactive decay. Plant samples were digested with concentrated HNO_3_–H_2_O_2_^18^ and the stable isotopes ^133^Cs and ^85^Rb were analyzed by inductively coupled plasma mass spectroscopy (ICP-MS; ELAN DRC-e, Perkin-Elmer Sciex). The concentrations of K^+^ and Na^+^ in plant samples were determined by inductively coupled plasma-optical emission spectroscopy (ICP-OES; 700 Series, Agilent Technologies) after digestion with HNO_3_–H_2_O_2_. Exchangeable K^+^ and Na^+^ were extracted from soil with 1 M CH_3_COONH_4_ (=1:10, pH 7.0) and analyzed by ICP-OES.

### Gene identification by using a combination of SNP linkage mapping and whole-genome resequencing

An F_2_ population was derived from a cross between *lcs1* and the *indica* rice cultivar Habataki. The F_2_ progeny consisting of 89 individuals and the parents were grown until grain filling under the same conditions as for the mutant screening. The concentrations of ^133^Cs and ^85^Rb in individual plants were analyzed by ICP-MS as described above. At the seedling stage, a piece of leaf blade was collected for genomic DNA extraction. To genotype the F_2_ population, 355 SNPs were selected from a world core SNP set, which includes 768 SNPs that are relatively evenly spaced on the 12 chromosomes^47^. SNPs were detected using the GoldenGate BeadArray technology platform and BeadStation 500 G system (Illumina) according to the manufacturer’s instructions. QTL analysis was conducted to map the candidate gene loci for *lcs1* according to Ishikawa *et al*.^17^. Five simple sequence repeat (SSR) markers were added to delimit the interval containing the candidate gene.

Whole-genome resequencing of WT and *lcs1* was performed according to Takagi *et al*.^48^. The libraries for DNA sequencing were constructed with a TruSeq DNA PCR-Free LT Sample Preparation Kit (Illumina) and sequenced on an Illumina NextSeq 500 platform (125 bp pared end sequence). Short reads were trimmed in 115 bp, and those in which >20% of the sequence had a Phred quality score of <20 were excluded from further analysis. Large deletions in *lcs1* were detected by comparing the sequence depth between WT and *lcs1*. To avoid library bias, three sequence libraries (n = 3) were constructed for WT and *lcs1* (Supplementary Table S4). Sequence reads obtained from each library were independently aligned to the Nipponbare genome (http://rapdb.dna.affrc.go.jp/download/irgsp1.html) by using BWA^49^, and the aligned data were converted to SAM/BAM files using SAMtools^50^. The average depth was calculated for the three WT libraries in regions longer than 15 bp where all WT libraries, but not *lcs1* libraries had at least an aligned sequence reads. The regions with an average depth of >5 in WT were defined as large deletions in *lcs1*. SNPs between WT and *lcs1* were detected by using MutMap pipeline version 1.4.4 (http://genome-e.ibrc.or.jp/home/bioinformatics-team/mutmap) using combined sequence reads the three WT and *lcs1* libraries, respectively. SNPs with the depth > 7 and SNP-index = 1 were selected.

To confirm the results of the whole-genome resequencing, we designed PCR primers (sosP1 and sosP2) corresponding to the region flanking the deletion site in the candidate gene (*OsSOS2*; Os06g0606000) in *lcs1* (Supplementary Table S5). PCR products obtained by amplification of genomic DNA were directly sequenced using the BigDye Terminator Cycle Sequencing Kit 3.1 (Invitrogen) in an ABI3130xl genetic analyzer (Applied Biosystems). The same primers were used to distinguish the WT and *lcs1* alleles.

### Analysis of *OsSOS2* transcript

Total RNA was extracted from whole roots of WT and *lcs1* plants using an RNeasy mini Kit (Qiagen) and quantified using a Nanodrop 8000 spectrophotometer (Thermo Scientific). First-strand cDNA was synthesized from 0.5 μg RNA with ReverTra Ace qPCR RT Master Mix with gDNA Remover (Toyobo) according to the manufacturers’ instructions. The transcript of each exon in *OsSOS2* was analyzed by RT-PCR using the primers (sosP3, sosP4, sosP5, and sosP6) listed in Supplementary Table S5.

### Physiological analysis of the *lcs1* mutant in hydroponics

Seeds of WT and *lcs1* were surface-sterilized and one-week-old seedlings were hydroponically cultured in a 1.3-L plastic pot filled with K^+^- and Na^+^-free modified 1/2 strength Kimura B nutrient solution^25^. Then, the seedlings were pre-cultured in the nutrient solution supplemented with 0.2 mM KCl for 5 days. In treatments, K^+^ was added to the nutrient solution as KCl, Na^+^ as NaCl, ^133^Cs^+^ as CsCl, and ^85^Rb^+^ as RbCl. To examine the Na^+^ sensitivity, pre-cultured WT and *lcs1* plants were treated with 100 mM Na^+^ in the presence of 0.2 mM K^+^ for 5 days. To examine the effect of different Na^+^ levels on Cs^+^ uptake, pre-cultured plants were treated for 24 h with varying levels of Na^+^ (0, 1, 5, 10, or 50 mM) in the presence of 0.1 μM Cs^+^ under low-K^+^ (0.05 mM) conditions. For the time-course Cs^+^ uptake experiment, pre-cultured plants were treated with 0.1 μM Cs^+^ in the presence or absence of 10 mM Na^+^ under low-K^+^ (0.05 mM) conditions for 1, 2, 4, or 6 days. To examine the effect of different K^+^ levels on Cs^+^ uptake, pre-cultured plants were treated with varying levels of K^+^ (0.02, 0.05, 0.2, or 1 mM) in the presence of 0.1 μM Cs^+^ plus 10 mM Na^+^ for 6 days. In the Rb^+^ uptake experiment, pre-cultured plants were treated with 0.1 μM Rb^+^ in the presence or absence of 10 mM Na^+^ under low-K^+^ (0.05 mM) conditions for 6 days. The pH of all solutions was adjusted to 5.0 with H_2_SO_4_ and NH_4_OH, and the solution was renewed every day. All plants were grown in a Biotron (NC350, NK System) with a 16-h-light (28 °C) /8-h-dark (25 °C) photoperiod, relative humidity of 60%, and light intensity of 400 μmol m^−2^ s^−1^. All plants were divided into shoots and roots, dried at 70 °C for 3 days, and weighed. The concentrations of K^+^, Na^+^, ^133^Cs, and ^85^Rb in shoots and roots were analyzed by ICP-OES or ICP-MS after digestion with HNO_3_–H_2_O_2_, as described above.

### Functional complementation test with the *Arabidopsis sos2-1* mutant and an F_2_ population from a cross between *lcs1* and *lcd-kmt2*

Seeds of *Arabidopsis* ecotype Columbia and the *sos2-1* mutant^21^ were surface-sterilized and sown on MS plates containing 0.8% (w/v) agar and 2% sucrose. Plates were placed in a growth chamber (EYELATRON FLI-301N, EYELA) with a 16-h-light (22 °C)/8-h-dark (22 °C) photoperiod and light intensity of 150 μmol m^−2^ s^−1^. Three-week-old plants were transferred into the nutrient solution described above. The solution contained 0.05 mM K^+^, 1 μM Cs^+^, and 1 μM Rb^+^ in the presence or absence of 10 mM NaCl (pH 5.5). The solution was renewed every day. The plants were harvested after 6 days, and the concentrations of ^133^Cs, ^85^Rb, K^+^, and Na^+^ in shoots and roots were analyzed by ICP-MS or ICP-OES after digestion with HNO_3_–H_2_O_2_, as described above.

To demonstrate that the *lcs1* allele confers a low-Cs phenotype on a cultivar carrying wild-type *OsSOS2* and to produce double mutants with low concentrations of both Cs^+^ and Cd, *lcs1* and *lcd-kmt2* (a low-Cd mutant)^17^ were crossed and the F_2_ seedlings (91 individuals) were pre-cultured in the nutrient solution as described above. A small leaf piece was collected from each seedling for genomic DNA extraction. Pre-cultured plants were treated with solution containing 1 µM Cs^+^ and 0.18 µM Cd^2+^ in the presence of 10 mM Na^+^ under low-K^+^ (0.05 mM) conditions for 3 days. The F_2_ population was genotyped by using genetic markers that can detect the *lcs1* (sosP1 and sosP2) and *lcd-kmt2* alleles^17^. The concentrations of ^133^Cs and ^114^Cd in shoots and roots were analyzed by ICP-MS after digestion with HNO_3_–H_2_O_2_ as described above.

### Microarray and qRT-PCR

For microarray analysis, pre-cultured WT and *lcs1* plants were transferred to the nutrient solution containing K^+^ (0.05 mM or 0.2 mM) in the presence or absence of 10 mM Na^+^ and grown for 3 days. Total RNA was isolated from whole roots using an RNeasy mini kit, quantified using Nanodrop, and its quality was checked using an Agilent 2100 bioanalyzer (Agilent Technologies). Microarray analyses were performed according to the manufacturer’s protocol and Yamatani *et al*.^51^. Briefly, RNA samples were labeled with Cy-3 and Cy-5 using a Quick Amp Labeling Kit, Two-Color (Agilent Technologies). A mixture of Cy3-labeled and Cy5-labeled cRNA (825 ng each) was fragmented and hybridized with the rice 4 × 44 K microarray RAP-DB (Agilent Technologies; G2519F#15241) at 65 °C for 17 h. Slides were washed with washing buffer, and scanned on an Agilent G2505B DNA microarray scanner. Background correction of the raw signals was performed using Agilent Feature Extraction software (version 9.5.3.1).

To confirm the differences in gene expression between WT and *lcs1* plants, qRT-PCR was performed. One-week-old seedlings were treated with 0.02 mM or 1 mM K^+^ for 7 days. Plants exposed to 0.02 mM K^+^ were then treated with 10 mM Na^+^ for 2, 6, 12, or 24 h. All samples were frozen immediately in liquid nitrogen and stored at −80 °C until RNA extraction. Total RNA was extracted and quantified as above. Its integrity was checked using agarose gel electrophoresis, and cDNA was synthesized from 0.5 μg RNA with ReverTra Ace qPCR RT Master Mix with gDNA Remover as described above. cDNA was diluted 1:8 in 1/10 TE buffer (1 mM Tris-HCl, 0.1 mM EDTA, pH 8.0). The reaction mixture contained 4 µL of diluted cDNA, 10 µL of 2 × SYBR Green PCR Master Mix (Toyobo), 1 µL of forward and reverse primers (final concentration, 0.3 µM), and 5 µL of PCR-grade H_2_O. Four reference genes, *Actin 1* (Os03g0718100), *Ubiquitin 5* (Os01g0328400), *β-tubulin 4* (Os01g0805900), and *Histone H3* (Os06g0130900) were tested to choose the most reliable reference gene for data normalization. The primers for *OsHAK1*, *OsHAK5*, *OsAKT1;1*, *OsHKT2;1*, and four reference genes are listed in Supplementary Table S5. Real-time PCR was performed on a LightCycler 96 (Roche) as follows: 1 min at 95 °C, then 40 cycles of 15 s at 95 °C and 1 min at 60 °C. The LightCycler 96 system software version 1.1.0.1320 was used to visualize and analyze the data including mean quantification cycle (Cq) values, amplification efficiencies, and melting curve. PCR efficiency was evaluated using a standard curve and was found to be 1.94 or 2.03 depending on the primer pairs. The expression stability and pairwise variation of the reference genes was analyzed using the geNorm algorithm in the qbase+ software (https://www.qbaseplus.com/), and *Actin 1* and *β-tubulin 4* were chosen as the best reference genes. Because *Actin 1* was most stably expressed during the treatments, we used it as an internal standard to normalize the expression data of the target genes. Five biological replicates per sample were used for qRT-PCR and two technical replicates were analyzed per biological replicate. The relative expression levels of target genes were evaluated using the 2^−ΔΔCq^ method according to Livak and Schmittgen^52^.

### Statistics

All values are presented as means ± standard deviation of the means (SD). Significance of the differences between WT and *lcs1* was analyzed by Student’s *t*-test using SPSS Statistics version 20.0 (IBM Japan). Significance levels (*P < 0.05 or **P < 0.01) and the number of replicates are given in each figure legend.

## Electronic supplementary material

Supplementary Information
