# Supplementary material for: Low-cesium rice: mutation in OsSOS2 reduces radiocesium in rice grains
Source: Sci Rep. 2017 May 25;7:2432. doi: 10.1038/s41598-017-02243-9 (PMC5445092; doi:10.1038/s41598-017-02243-9)
Supplement: Supplementary file 1 — Supplementary Information [file 41598_2017_2243_MOESM1_ESM.doc]

**Supplementary information**

**Low-cesium rice: mutation in *OsSOS2* reduces radiocesium in rice grains**

Satoru Ishikawa^1^,*, Shimpei Hayashi^1,2^, Tadashi Abe^1^, Masato Igura^1^, Masato Kuramata^1^, Hachidai Tanikawa^1^, Manaka Iino^1^, Takashi Saito^3^, Yuji Ono^4^, Tetsuya Ishikawa^5^, Shigeto Fujimura^5^, Akitoshi Goto^6^ & Hiroki Takagi^7,8^

^1^ Institute for Agro-Environmental Sciences, National Agriculture and Food Research Organization (NARO), Tsukuba 305-8604, Japan.

^2^ Institute of Agrobiological Sciences, NARO, Tsukuba 305-8604, Japan.

^3^ Hama Agricultural Regeneration Research Centre, Fukushima Agricultural Technology Centre, Minamisoma 975-0007, Japan.

^4^ Fruit Tree Research Centre, Fukushima Agricultural Technology Centre, Fukushima 960-0231, Japan.

^5^ Tohoku Agricultural Research Centre, NARO, Fukushima 960-2156, Japan.

^6^ Institute of Crop Science, NARO, Tsukuba 305-8518, Japan.

^7^ Iwate Biotechnology Research Center, Kitakami 024-0003, Japan.

*To whom correspondence should be addressed. E-mail: [isatoru@affrc.go.jp](mailto:isatoru@affrc.go.jp)

**
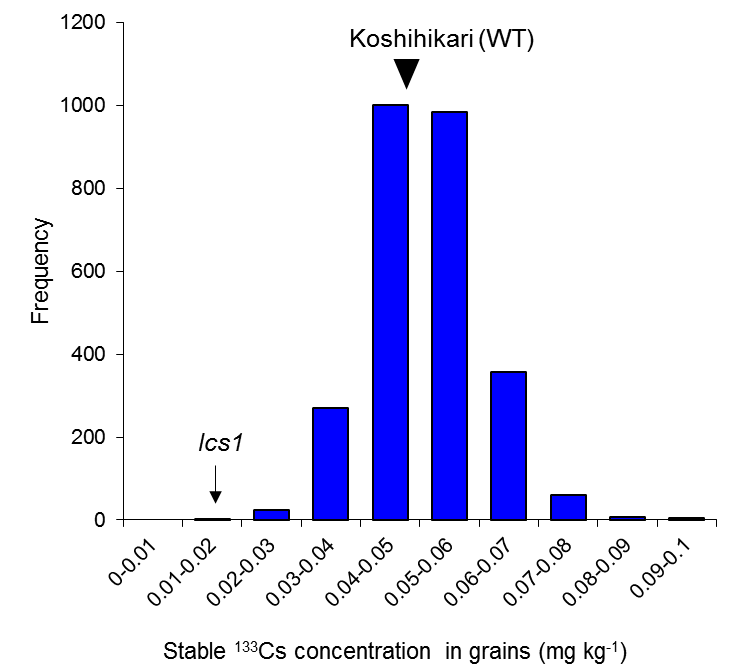
**

**Supplementary Figure S1.** Frequency distribution of grain ^133^Cs concentration in rice mutants (2710 M_2_ plants) grown in pots filled with paddy soil.

**
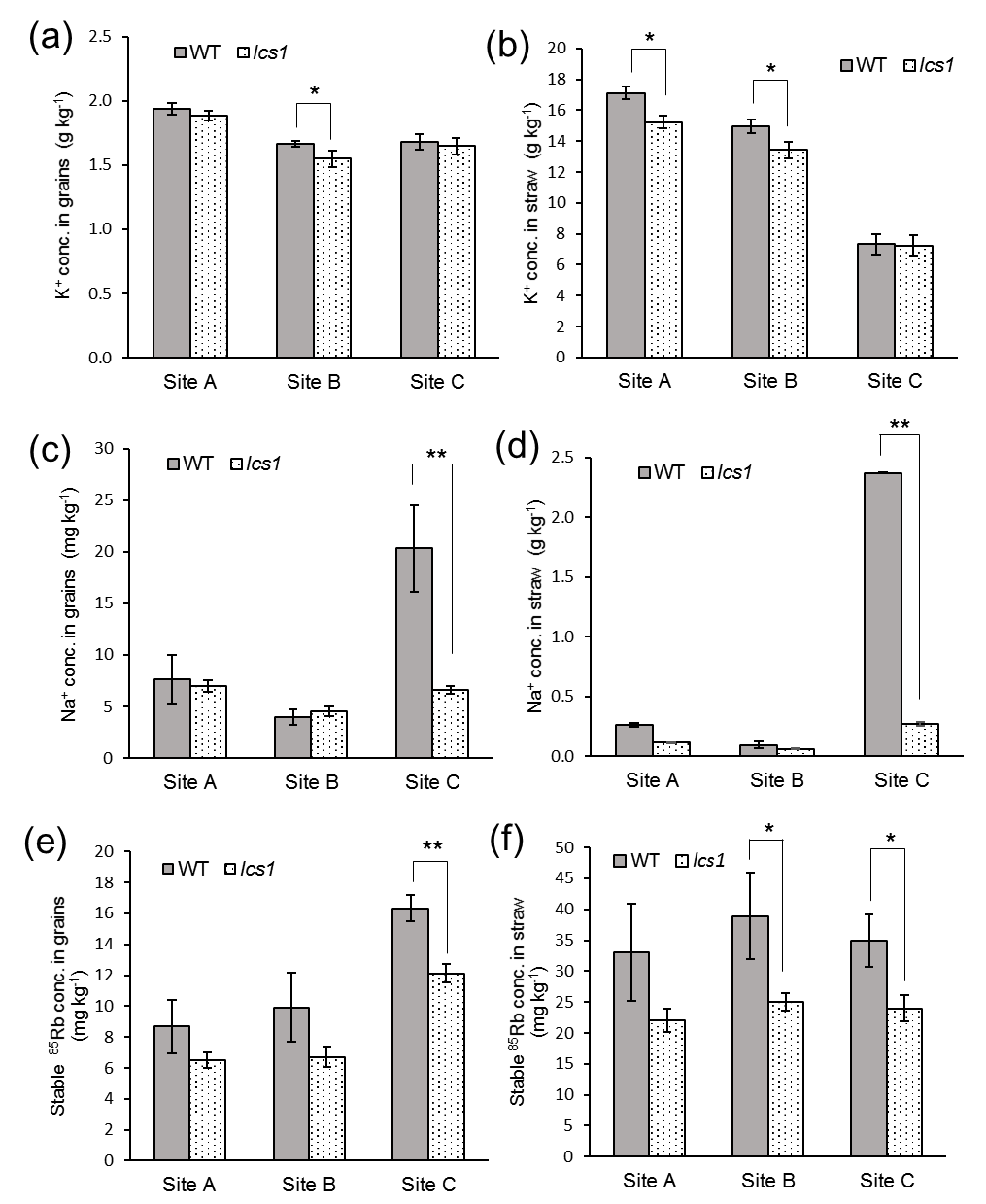
**

**Supplementary Figure S2.** Concentrations of K^+^, Na^+^, and ^85^Rb in Koshihikari (WT) and a rice mutant (*lcs1*) grown in three paddy fields located in Fukushima Prefecture. (a, b) K^+^ concentration in grains (a) and straw (b). (c, d) Na^+^ concentration in grains (c) and straw (d). (e, f) ^85^Rb concentration in grains (e) and straw (f). Data are presented as means ± SD of three biological replicates. ** *P* < 0.01, * *P* < 0.05 (*t*-test).

**
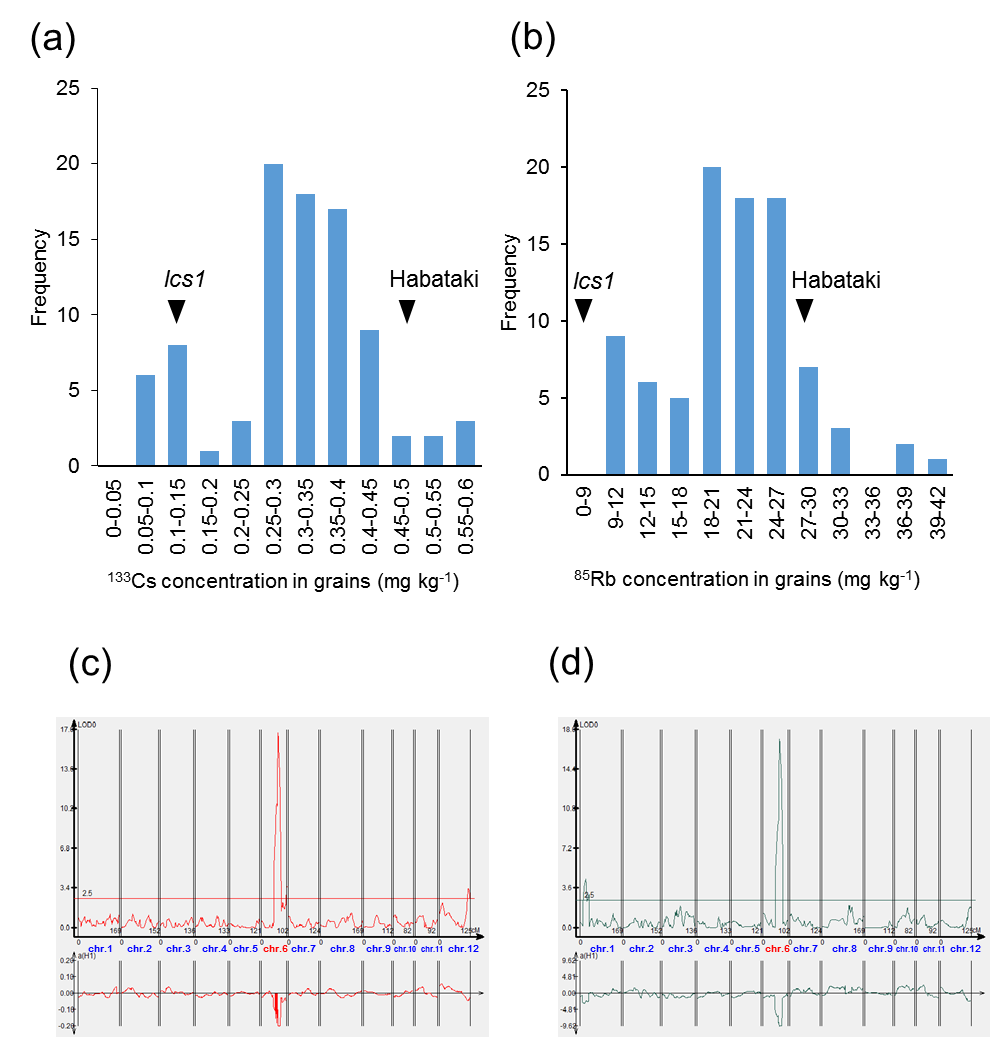
**

Continued

**
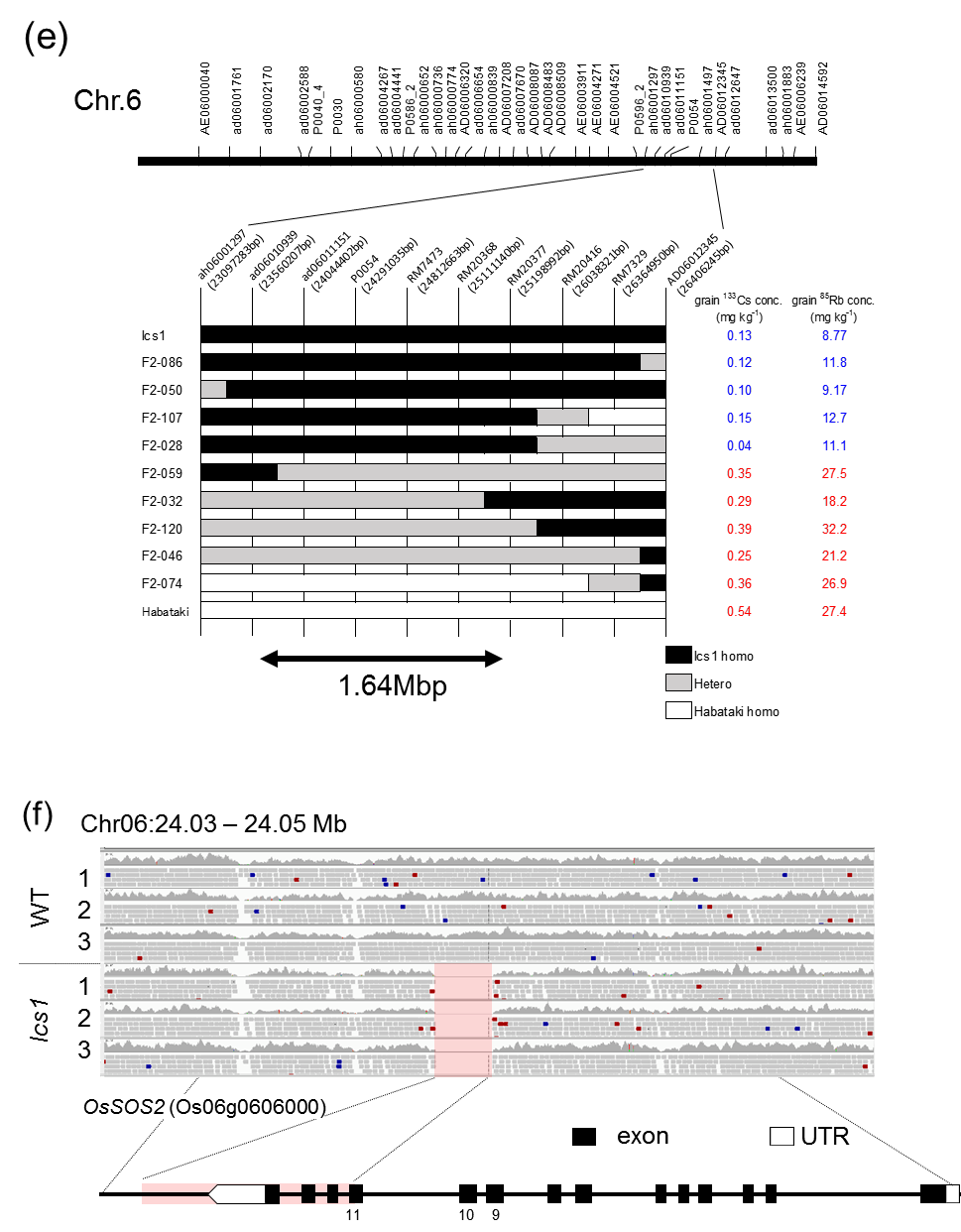
**

**Supplementary Figure S3.** Positional cloning of the gene responsible for the decreased Cs^+^ uptake in *lcs1*. (a, b) Frequency distributions of grain Cs (a) and Rb (b) concentrations in 89 F_2_ plants derived from a cross between *lcs1* and the *indica* cultivar Habataki. (c, d) Logarithm of odds (LOD) curves for Cs (c) and Rb (d) in grain. (e) Linkage analysis to map a QTL for low Cs and Rb concentrations on chromosome 6. (f) A snapshot of an IGV genome viewer window showing the location of the *OsSOS2* gene in the 24.03–24.05 Mb region of chromosome 6 in the Nipponbare reference genome. Aligned short reads (three replications of resequencing) are shown for WT and *lcs1*. The red shadow indicates the identified large deletion in *lcs1*.


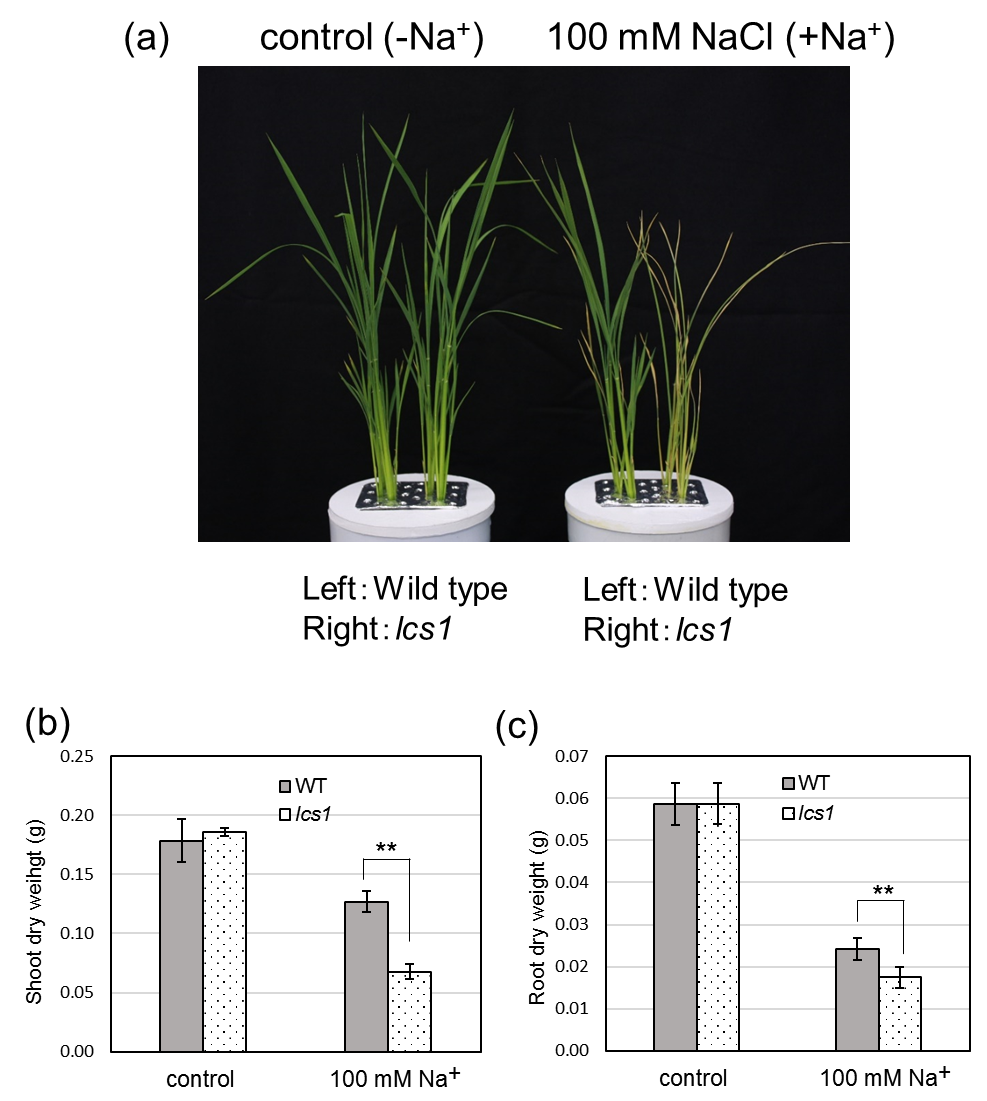


**Supplementary Figure S4.** Effects of Na^+^ stress on the *lcs1* mutant. (a) Phenotypes of wild-type Koshihikari and *lcs1* treated with 100 mM Na^+^ or without Na^+^ (control). (b, c) Dry weight of shoots (b) and roots (c) in the WT and *lcs1* grown in the absence of Na^+^ (control) or in the presence of 100 mM Na^+^. Data are means ± SD of four biological replicates. ** *P* < 0.01 (*t*-test).

**
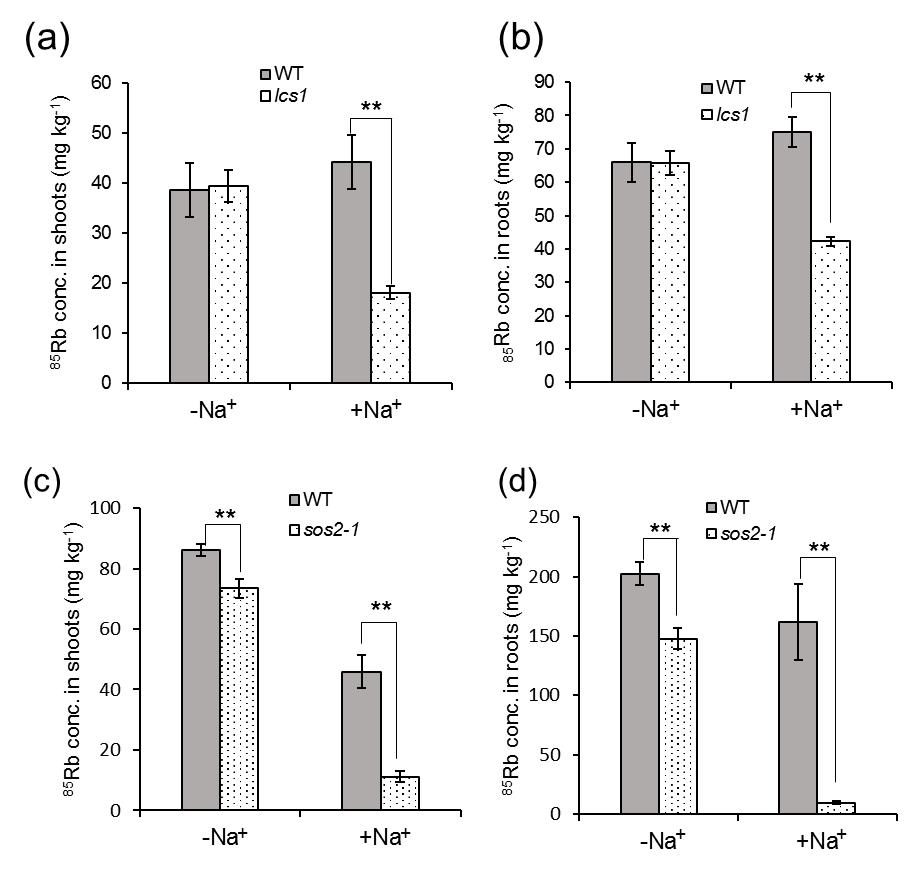
**

**Supplementary Figure S5.** Concentrations of stable ^85^Rb in shoots and roots of rice (Koshihikari and *lcs1*) and *Arabidopsis thaliana* (Columbia and *sos2-1*). Plants were treated with nutrient solution containing 0.1 µM Rb^+^ (rice) or 1µM Rb^+^ (*A. thaliana*) in the presence (+Na^+^) or absence (−Na^+^) of 10 mM Na^+^ under low-K^+^ (0.05 mM) conditions. (a, b) Rice. (c, d) *A. thaliana*. Data are means ± SD of three biological replicates. ** *P* < 0.01 (*t*-test).

**
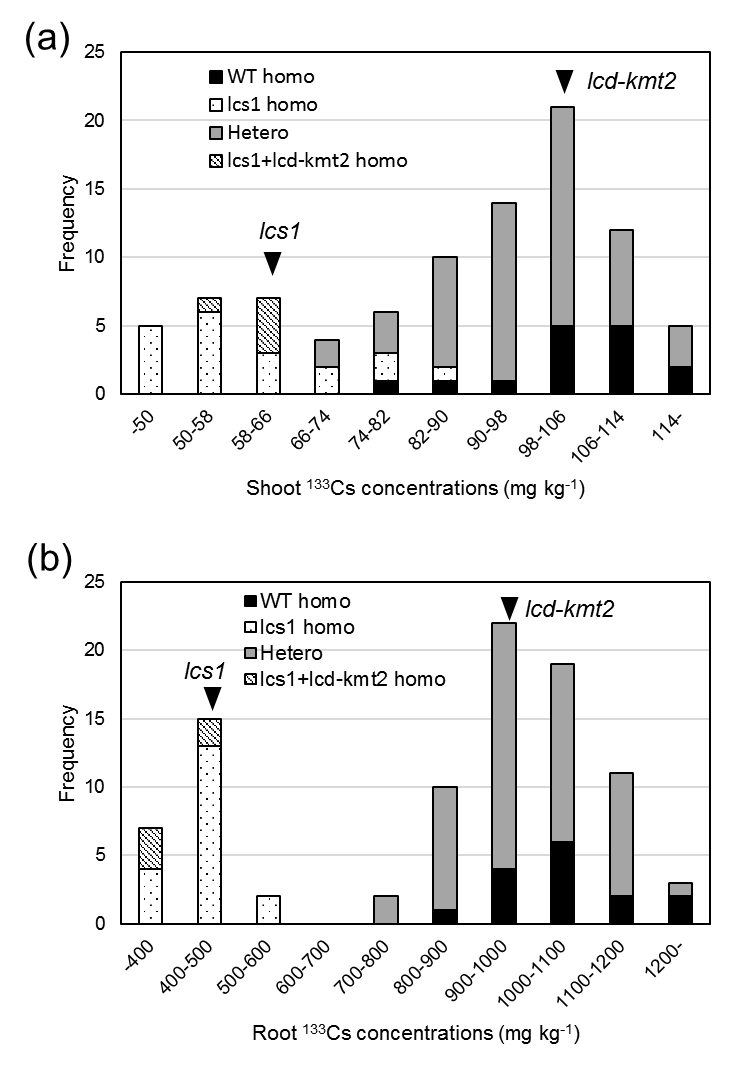
**

**Supplementary Figure S6.** Frequency distribution of Cs concentrations in (a) shoots and (b) roots of F_2_ plants derived from a cross between *lcs1* and *lcd-kmt2*, a low-Cd mutant. Using DNA markers that detect the *lcs1* allele or the *lcd-kmt2* allele, 91 F_2_ plants were classified into four genotype classes: homozygous for the *OsSOS2* allele (WT homo), homozygous for the *lcs1* allele (*lcs1* homo), heterozygous (*OsSOS2* and *lcs1*), and homozygous for both *lcs1* and *lcd-kmt2*.

**Supplementary Table S1.** Soil properties in three experimental fields.

| Site | Soil type | ^134^Cs | ^137^Cs | Exchangeable K^+^ | Exchangeable Na^+^ | K^+^/Na^+^ ratio |
| --- | --- | --- | --- | --- | --- | --- |
|  |  | (Bq kg^−1^) | (Bq kg^−1^) | (mg kg^−1^) | (mg kg^−1^) |  |
| A | Typic Fluvaquent | 1057 | 4721 | 115.4 | 30.3 | 3.81 |
| B | Typic Fluvaquent | 1058 | 3894 | 64.4 | 34.7 | 1.85 |
| C | Hydric Melanaquand | 1705 | 6387 | 30.0 | 25.6 | 1.17 |

**Supplementary Table S2.** Mutations in the candidate region on chromosome 6 identified by whole-genome re-sequencing.

| Position | Mutation type | Putative gene | Gene annotation | Mutated region |
| --- | --- | --- | --- | --- |
| 24038611–24040095 | Deletion (1485 bp) | Os06g0606000 | Similar to CBL-interacting serine/threonine-protein kinase 24 (EC 2.7.1.37) (SNF1-related kinase 3.11) (SALT OVERLY SENSITIVE 2 protein) | Exons 11, 12, 13, 14, and 3′-UTR |
| 24589115 | SNP (A→C) | No hit |  |  |

**Supplementary Table S3.** Comparison of gene expression between the WT and *lcs1* mutant using microarray analysis.

| Gene ID | Gene name | 0.05 mM K^+^ for 3 days | | |  | 0.05 mM K^+^ + 10 mM Na^+^ for 3 days | | |  | 0.2 mM K^+^ for 3 days | | |  | 0.2 mM K^+^ + 10 mM Na  for 3 days | | |
| --- | --- | --- | --- | --- | --- | --- | --- | --- | --- | --- | --- | --- | --- | --- | --- | --- |
|  |  | Fold change | Signal (WT) | Signal (*lcs1*) |  | Fold change | Signal  (WT) | Signal  (*lcs1*) |  | Fold change | Signal (WT) | Signal (*lcs1*) |  | Fold change | Signal (WT) | Signal (*lcs1*) |
| Os01g0930400 | OsHAK5 | 0.70 | 205 | 144 |  | 0.59 | 108 | 64 |  | 0.44 | 92 | 40 |  | 0.45 | 126 | 56 |
| Os01g0935500 | OsHAK2 | 1.00 | 577 | 575 |  | 1.07 | 755 | 810 |  | 1.09 | 485 | 528 |  | 1.06 | 523 | 553 |
| Os03g0575200 | OsHAK16 | 1.04 | 106 | 110 |  | 1.70 | 152 | 259 |  | 0.78 | 136 | 106 |  | 0.79 | 112 | 89 |
| Os04g0401700 | OsHAK1 | 1.15 | 39007 | 44776 |  | 0.89 | 26503 | 23684 |  | 1.08 | 24498 | 26360 |  | 1.02 | 20310 | 20766 |
| Os09g0376900 | OsHAK23 | 1.13 | 3224 | 3654 |  | 0.89 | 3947 | 3528 |  | 0.98 | 2477 | 2432 |  | 0.97 | 2544 | 2465 |
| Os09g0448200 | OsHAK17 | 5.01 | 52 | 260 |  | 2.50 | 115 | 288 |  | 1.21 | 86 | 104 |  | 1.82 | 69 | 126 |
| Os09g0563200 | OsHAK18 | 1.26 | 857 | 1079 |  | 0.95 | 996 | 948 |  | 1.21 | 664 | 803 |  | 1.16 | 719 | 834 |
| Os01g0369300 | OsHAK3 | 0.65 | 7 | 5 |  | 0.54 | 8 | 4 |  | 0.76 | 6 | 4 |  | 0.50 | 8 | 4 |
| Os01g0932500 | OsHAK6 | 0.44 | 7 | 3 |  | 2.02 | 3 | 6 |  | 0.41 | 4 | 2 |  | 0.44 | 4 | 2 |
| Os02g0518600 | OsHAK19 | 0.91 | 6 | 6 |  | 3.03 | 4 | 13 |  | 1.77 | 3 | 5 |  | 2.09 | 3 | 7 |
| Os02g0519100 | OsHAK20 | 0.28 | 7 | 2 |  | 0.53 | 5 | 3 |  | 0.29 | 11 | 3 |  | 0.67 | 6 | 4 |
| Os02g0730300 | OsHAK25 | 1.05 | 127 | 133 |  | 0.87 | 244 | 212 |  | 1.19 | 143 | 170 |  | 1.00 | 153 | 154 |
| Os03g0337500 | OsHAK8 | 0.80 | 34 | 28 |  | 0.83 | 45 | 37 |  | 0.67 | 34 | 23 |  | 0.69 | 47 | 33 |
| Os03g0574900 | OsHAK27 | 0.61 | 3 | 2 |  | 0.73 | 4 | 3 |  | 0.74 | 3 | 2 |  | 0.68 | 3 | 2 |
| Os04g0610700 | OsHAK15 | 0.91 | 2565 | 2323 |  | 0.81 | 3063 | 2486 |  | 0.93 | 2145 | 1985 |  | 0.98 | 1989 | 1959 |
| Os04g0613900 | OsHAK11 | 1.00 | 302 | 301 |  | 0.91 | 439 | 399 |  | 0.94 | 331 | 313 |  | 1.08 | 297 | 320 |
| Os06g0270200 | OsHAK24 | 1.17 | 449 | 526 |  | 1.00 | 799 | 798 |  | 1.01 | 674 | 678 |  | 1.18 | 673 | 797 |
| Os06g0625900 | OsHAK10 | 0.82 | 5272 | 4319 |  | 0.86 | 4749 | 4093 |  | 0.95 | 3562 | 3372 |  | 1.16 | 3595 | 4168 |
| Os06g0671000 | OsHAK13 | 0.91 | 65 | 60 |  | 0.96 | 112 | 107 |  | 1.20 | 66 | 79 |  | 1.17 | 79 | 92 |
| Os07g0102100 | OsHAK22 | 0.93 | 22 | 21 |  | 1.24 | 25 | 30 |  | 0.87 | 22 | 19 |  | 0.95 | 36 | 34 |
| Os07g0509200 | OsHAK14 | 0.74 | 387 | 285 |  | 1.02 | 355 | 363 |  | 0.85 | 237 | 201 |  | 0.90 | 293 | 264 |
| Os07g0669700 | OsHAK7 | 1.07 | 19561 | 20932 |  | 0.62 | 24816 | 15327 |  | 1.01 | 10886 | 10944 |  | 0.98 | 14919 | 14580 |
| Os07g0679000 | OsHAK9 | 1.00 | 476 | 478 |  | 0.94 | 507 | 476 |  | 1.00 | 337 | 337 |  | 1.26 | 331 | 418 |
| Os08g0466200 | OsHAK4 | 0.91 | 286 | 261 |  | 1.43 | 223 | 318 |  | 1.28 | 201 | 257 |  | 0.92 | 322 | 295 |
| Os08g0510300 | OsHAK26 | 0.59 | 4 | 2 |  | 0.71 | 4 | 3 |  | 0.73 | 3 | 2 |  | 0.65 | 4 | 2 |
| Os01g0648000 | OsAKT1.1 | 0.94 | 4802 | 4536 |  | 0.52 | 4901 | 2536 |  | 1.18 | 2754 | 3251 |  | 0.66 | 2753 | 1813 |
| Os05g0428700 | OsAKT2 | 0.86 | 753 | 646 |  | 0.55 | 427 | 235 |  | 0.91 | 267 | 242 |  | 0.58 | 282 | 164 |
| Os07g0175400 | OsAKT1.2 | 0.49 | 6 | 3 |  | 0.57 | 11 | 6 |  | 0.91 | 4 | 3 |  | 0.79 | 8 | 6 |
| Os01g0307500 | OsHKT1.5 | 1.77 | 43 | 77 |  | 1.68 | 122 | 205 |  | 1.08 | 290 | 312 |  | 1.25 | 227 | 283 |
| Os01g0532600 | OsHKT2.3 | 0.62 | 4 | 3 |  | 0.71 | 5 | 3 |  | 0.72 | 3 | 2 |  | 0.64 | 4 | 3 |
| Os02g0175000 | OsHKT1.3 | 0.58 | 3 | 2 |  | 7.38 | 4 | 33 |  | 1.31 | 5 | 6 |  | 1.55 | 7 | 12 |
| Os04g0607500 | OsHKT1.1 | 0.91 | 49 | 45 |  | 0.68 | 30 | 21 |  | 1.55 | 11 | 18 |  | 1.33 | 13 | 18 |
| Os04g0607600 | OsHKT1.4 | 1.70 | 13 | 23 |  | 0.32 | 8 | 3 |  | 0.72 | 4 | 3 |  | 0.66 | 3 | 2 |
| Os06g0701600 | OsHKT2.4 | 0.73 | 3 | 2 |  | 1.18 | 3 | 4 |  | 1.12 | 4 | 4 |  | 0.69 | 3 | 2 |
| Os06g0701700 | OsHKT2.1 | 1.06 | 4014 | 4239 |  | 0.33 | 438 | 147 |  | 1.47 | 164 | 242 |  | 0.95 | 83 | 79 |
| Os09g0558300 | OsCNGC9 | 1.03 | 3898 | 4033 |  | 1.09 | 4708 | 5134 |  | 1.12 | 3272 | 3659 |  | 1.21 | 3511 | 4260 |
| Os12g0163000 | OsCNGC8 | 0.64 | 6 | 4 |  | 0.56 | 9 | 5 |  | 1.19 | 7 | 8 |  | 0.70 | 3 | 2 |
| Os12g0468500 | OsCNGC5 | 0.79 | 22 | 18 |  | 0.86 | 26 | 22 |  | 0.76 | 16 | 12 |  | 0.80 | 20 | 16 |
| Os01g0782800 | OsCNGC15 | 0.66 | 3 | 2 |  | 0.78 | 4 | 3 |  | 0.59 | 3 | 2 |  | 0.60 | 3 | 2 |
| Os02g0255000 | OsCNGC1 | 0.64 | 52 | 33 |  | 0.55 | 71 | 39 |  | 0.61 | 45 | 28 |  | 0.84 | 46 | 38 |
| Os02g0627700 | OsCNGC7 | 3.32 | 3 | 10 |  | 0.75 | 3 | 2 |  | 0.73 | 2 | 2 |  | 0.69 | 3 | 2 |
| Os02g0773400 | OsCNGC12 | 0.98 | 309 | 304 |  | 1.04 | 341 | 354 |  | 0.98 | 279 | 273 |  | 0.99 | 281 | 279 |
| Os02g0789100 | OsCNGC10 | 0.86 | 823 | 705 |  | 0.79 | 1067 | 846 |  | 0.89 | 715 | 636 |  | 0.92 | 770 | 708 |
| Os03g0646300 | OsCNGC4 | 0.81 | 122 | 99 |  | 0.91 | 139 | 126 |  | 1.19 | 63 | 75 |  | 1.30 | 96 | 125 |
| Os03g0758300 | OsCNGC14 | 1.29 | 767 | 993 |  | 0.47 | 1329 | 620 |  | 1.09 | 674 | 733 |  | 0.65 | 935 | 603 |
| Os04g0643600 | OsCNGC6 | 1.06 | 3688 | 3904 |  | 1.10 | 4875 | 5361 |  | 1.11 | 3072 | 3414 |  | 1.21 | 3274 | 3958 |
| Os05g0502000 | OsCNGC16 | 0.58 | 3 | 2 |  | 0.43 | 7 | 3 |  | 0.69 | 3 | 2 |  | 0.66 | 3 | 2 |
| Os06g0188000 | OsCNGC11 | 1.10 | 3096 | 3392 |  | 1.03 | 4126 | 4236 |  | 1.10 | 2282 | 2512 |  | 1.11 | 2903 | 3217 |
| Os06g0207700 | OsCNGC13 | 1.19 | 433 | 514 |  | 1.20 | 310 | 371 |  | 1.02 | 267 | 273 |  | 1.00 | 275 | 275 |
| Os06g0527100 | OsCNGC2 | 1.02 | 1930 | 1969 |  | 0.91 | 2363 | 2147 |  | 0.96 | 1857 | 1776 |  | 1.08 | 1747 | 1886 |

**Supplementary Table S4. Summary of the sequence reads from Illumina NextSeq500 sequencing.**

| Sample^a^ | Replication | Number of reads | Read length (bp) | Total sequence (Gb) | Genome coverage (%)^b^ | Mean depth^c^ |
| --- | --- | --- | --- | --- | --- | --- |
| WT | 1 | 14,225,483 | 115 | 3.27186109 | 0.984 | 14.874 |
|  | 2 | 20,404,972 | 115 | 4.69314356 | 0.981 | 10.148 |
|  | 3 | 17,098,422 | 115 | 3.93263706 | 0.984 | 14.325 |
| *lcs1* | 1 | 24,928,006 | 115 | 5.73344138 | 0.978 | 8.567 |
|  | 2 | 16,866,748 | 115 | 3.87935204 | 0.982 | 12.180 |
|  | 3 | 23,970,598 | 115 | 5.51323754 | 0.981 | 10.244 |

^a^For each sample, sequence reads were aligned to the Nipponbare genome. Sequence reads were trimmed to 115 bp, and those in which >20% of the sequence had a Phred quality score <20 were excluded.

^b^Percentage of the total Nipponbare genome overlapping with short reads.

^c^Average read depth over the whole genome.

**Supplementary Table S5.** Primers used in this study.

| Primer | Sequence (5′–3′) |
| --- | --- |
| sosP1 | TTTGTGCTCTTACATTAGATCTTCCTAGAC |
| sosP2 | AAAGGCTTCTTTTACCGTTCTGTGTACCTG |
| sosP3 | GGCTGTTTTTGACAATATTGAGGAC |
| sosP4 | CTGCTGTCTATCAAATAATGCTG |
| sosP5 | GCTGGTTTCCTTGAGACAAAACGAG |
| sosP6 | GCCACTAATTTGATTATGGATTGGAAGG |
| Actin 1 qRT F | GCGTGGACAAAGTTTTCAACCG |
| Actin 1 qRT R | TCTGGTACCCTCATCAGGCATC |
| Ubiquitin 5 qRT F | CAACCAGCAGGCTTAGGCGTAG |
| Ubiquitin 5 qRT R | GGTGTTCAGTTCCAAGGAGAC |
| β-tubulin 4 qRT F | CAGTACCGTGCCCTTACTGTTCC |
| β-tubulin 4 qRT R | CTCTCGGTGGAATGTCACAGACAC |
| Histone H3 qRT F | AGTTTGGTCGCTCTCGATTTCG |
| Histone H3 qRT R | TCAACAAGTTGACCACGTCACG |
| OsHAK1 qRT F | GTTGATGATGCTGATGTTGGAAGAG |
| OsHAK1 qRT R | TTACACAAACCACACTGTACAGAG |
| OsHAK5 qRT F | GATGTTGTTCTCAGTGCTGAGTG |
| OsHAK5 qRT R | GTTCTGTGGTATGGTCAGGATTAG |
| OsAKT1 qRT F | CAGTAATGAATGGGATGCAGAG |
| OsAKT1 qRT R | CCCCTTTCTTGGAAATCAACAG |
| OsHKT2;1 qRT F | TCCATCGACTGCTCACTCA |
| OsHKT2;1 qRT R | TGTTGTCGATGGTGGTAAGTACA |
